# Supplementary material for: Theranostic Application of a Novel G-Quadruplex-Forming DNA Aptamer Targeting Malate Synthase of Mycobacterium tuberculosis
Source: Mol Ther Nucleic Acids. 2019 Oct 4;18:661–72. doi: 10.1016/j.omtn.2019.09.026 (PMC6849348; doi:10.1016/j.omtn.2019.09.026)
Supplement: Document S1. Supplemental Materials and Methods, Figures S1–S6, and Tables S1–S3 [file mmc1.pdf]

OMTN, Volume *18*

Supplemental Information

Theranostic Application of a Novel G-Quadruplex-Forming DNA Aptamer Targeting Malate Synthase of Mycobacterium tuberculosis

Abhijeet Dhiman, Chanchal Kumar, Subodh Kumar Mishra, Kriti Sikri, Ishara Datta, Pradeep Sharma, Tej P. Singh, Sagarika Haldar, Neera Sharma, Anjali Bansal, Yusra Ahmad, Amit Kumar, Tarun Kumar Sharma, and Jaya Sivaswami Tyagi

SUPPLEMENTARY FIGURELEGENDS

Figure S1

Sequence alignment based on primary sequence homology reveals two categories i.e. G-rich and non-G rich group.

Figure S2

Base fraction analysis of selected aptamer candidates showing abundance of various base fractions (A, T, G or C), 50% of aptamer pool represents G-richness **(A)**and abundance of G+C and A+T **(B).**

Figure S3

Secondary structure of selected aptamer candidates predicted by web-based tool RNA-fold (http://rna.tbi.univie.ac.at/cgi-bin/RNAWebSuite/RNAfold.cgi) using DNA parameters.

Figure S4

The SPR-sensograms for the bindings of ssDNA aptamers namely, MS4, MS5, MS6, MS10, MS20, MS10-Trunc and R0 (Naïve library) with malate synthase (MS). The protein MS was immobilized on the CM5sensor chip and the increasing concentrations (25, 50, 100, 200, 400 and 800 nM) of ssDNA aptamers were used in mobile phase in separate experiments.

Figure S5

Secondary structure of MS10 and its truncated form MS10-Trunc predicted by web-based tool RNA-fold (http://rna.tbi.univie.ac.at/cgi-bin/RNAWebSuite/RNAfold.cgi).

Figure S6

MS10-Trunc aptamer-based detection of Mtb MS in CSF specimens by ALISA. **(A)** Scatter plot of ΔOD450 value was generated from ‘Definite TB’ (True positive) vs. NTIM group (True negative). *** represent statistical significance at p value of <0.0001. **(B)** ROC curve generated from ‘Definite TB’ vs. ‘NTIM group’. The blue horizontal line across the scatter plot indicates the cut-off (0.3444) derived by ROC curve analysis of ‘Definite TB’ and NTIM disease specimens.

Classification of cerebrospinal fluid (CSF) samples

A subset of 86 pediatric CSF samples that was derived from an archived set of samples described previously by Haldar et al[1] was used in the present study. The samples were categorized according to a universal case definition for tuberculous meningitis (TBM)[2]. Culture/smear/commercial nucleic acid amplification test (NAAT) positive/ acid-fast bacillus (AFB) seen on autopsy were categorized as "definite" TBM. "Probable" and "possible" TBM groups include subjects negative by the above criteria but satisfying the defined clinical criteria, CSF biochemistry and cytology, cerebral imaging criteria and evidence of extraneural TB having a score of ≥10–12 ("probable" TBM) and a score of ≥6–11 ("possible" TBM). In this CSF sample subset (N=86), samples were classified as "definite" TBM on the basis of *M. tuberculosis* culture positivity only (N=16) and as "probable" TBM (N=6, score range: 10–18), "possible" TBM (N=16, score range: 6–9) and "not-tuberculous meningitis" (not-TBM) with an alternative diagnosis established (N=48). The "not-TBM" category was further sub-classified into nontuberculous infectious meningitis (NTIM, N=16), infectious neurological disorders (IND, N=17) and noninfectious neurological disorders (NIND, N=15). The NTIM comprised of cases of pyogenic bacterial meningitis that included 2 culture-confirmed cases of *E. coli* (N=1), and *Acinetobacter* sp. (N=1). Another 14 cases were diagnosed on the basis of response to appropriate antibiotics, clinical presentation and symptoms. The IND category included 6 cases of meningoencephalitis, 4 cases of enteric encephalopathy, 3 cases of sepsis, 2 cases of cerebral malaria and 1 case each of pneumonia and post diphtheritic polyneuritis. The NIND category included 8 cases of neurodegenerative disorders, 2 cases each of hypocalcemic seizures and transverse myelitis, 3 case of Guillain–Barré syndrome.

References

1 Haldar, S. *et al.* Detection of Mycobacterium tuberculosis GlcB or HspX Antigens or devR DNA impacts the rapid diagnosis of tuberculous meningitis in children. *PloS one* **7**, e44630, doi:10.1371/journal.pone.0044630 (2012).

2 Marais, S. *et al.* Tuberculous meningitis: a uniform case definition for use in clinical research. *The Lancet. Infectious diseases* **10**, 803-812, doi:10.1016/S1473-3099(10)70138-9 (2010).

3 Phan, A. T. *et al.* An interlocked dimeric parallel-stranded DNA quadruplex: a potent inhibitor of HIV-1 integrase. *Proceedings of the National Academy of Sciences of the United States of America* **102**, 634-639, doi:10.1073/pnas.0406278102 (2005).

4 Nagatoishi, S., Isono, N., Tsumoto, K. & Sugimoto, N. Loop residues of thrombin-binding DNA aptamer impact G-quadruplex stability and thrombin binding. *Biochimie* **93**, 1231-1238, doi:10.1016/j.biochi.2011.03.013 (2011).

5 Platella, C., Riccardi, C., Montesarchio, D., Roviello, G. N. & Musumeci, D. G-quadruplex-based aptamers against protein targets in therapy and diagnostics. *Biochimica et biophysica acta. General subjects* **1861**, 1429-1447, doi:10.1016/j.bbagen.2016.11.027 (2017).

Supplementary Figure S1

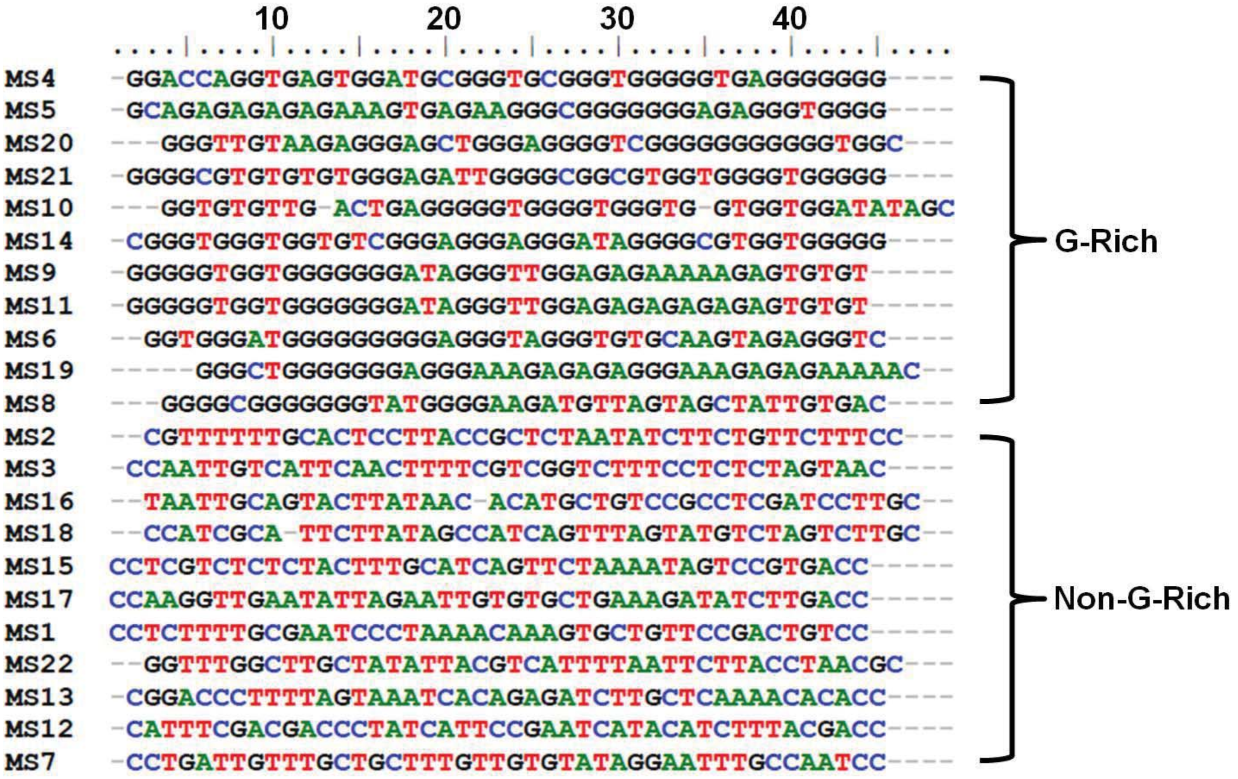

Supplementary Figure S2

A

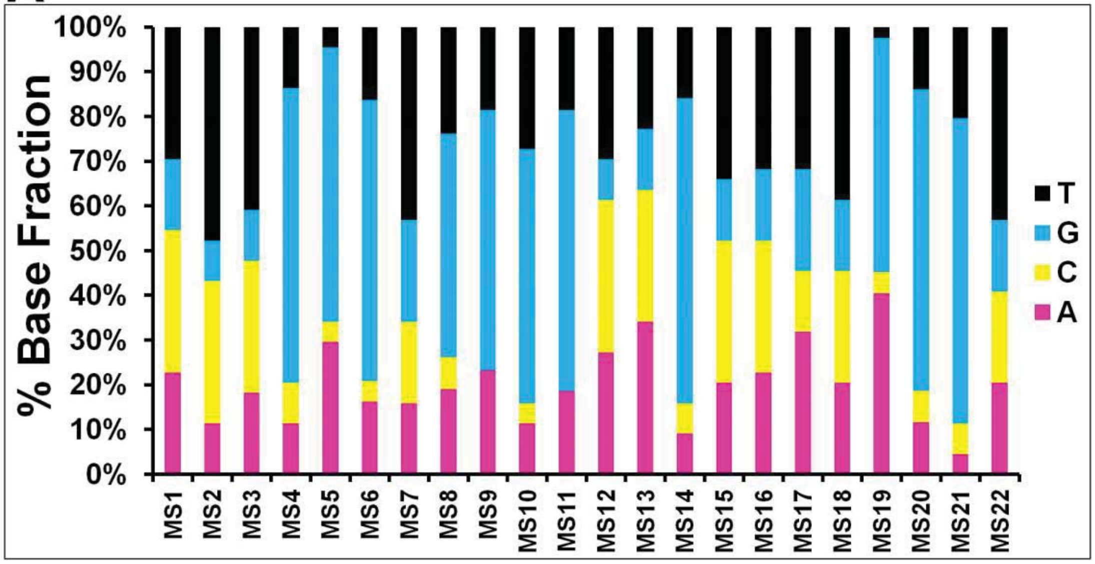

B

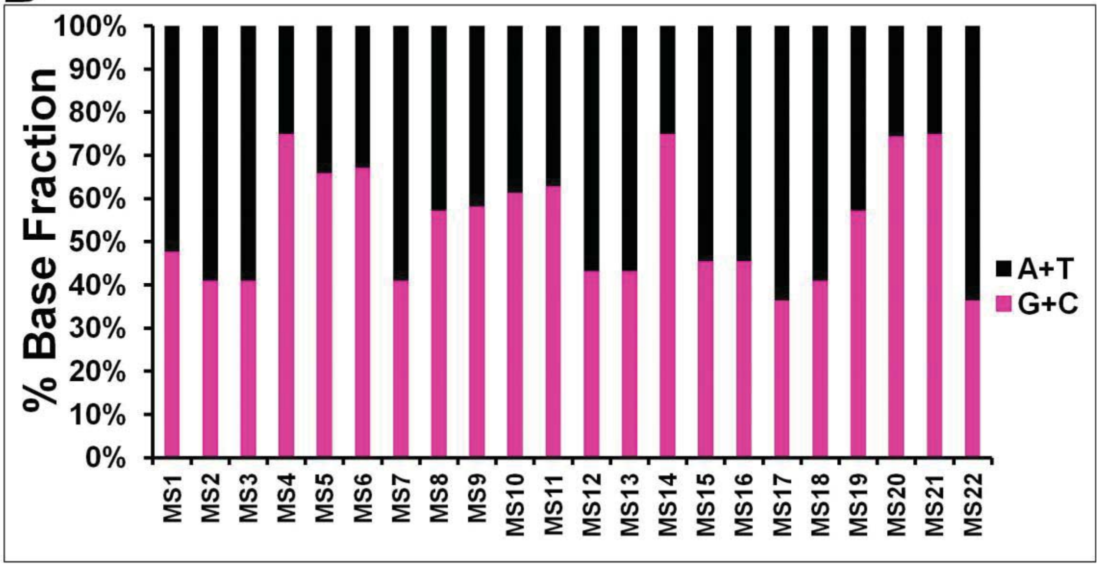

Supplementary Figure S3

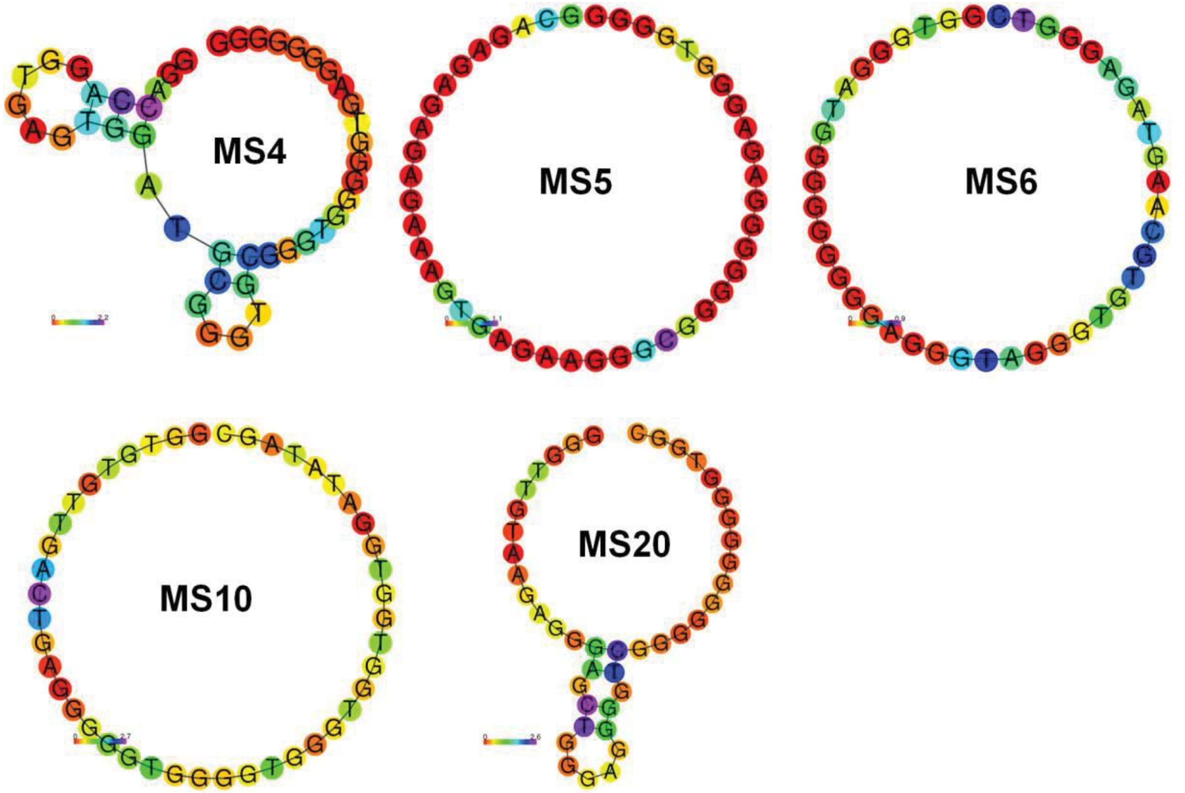

Supplementary Figure S4

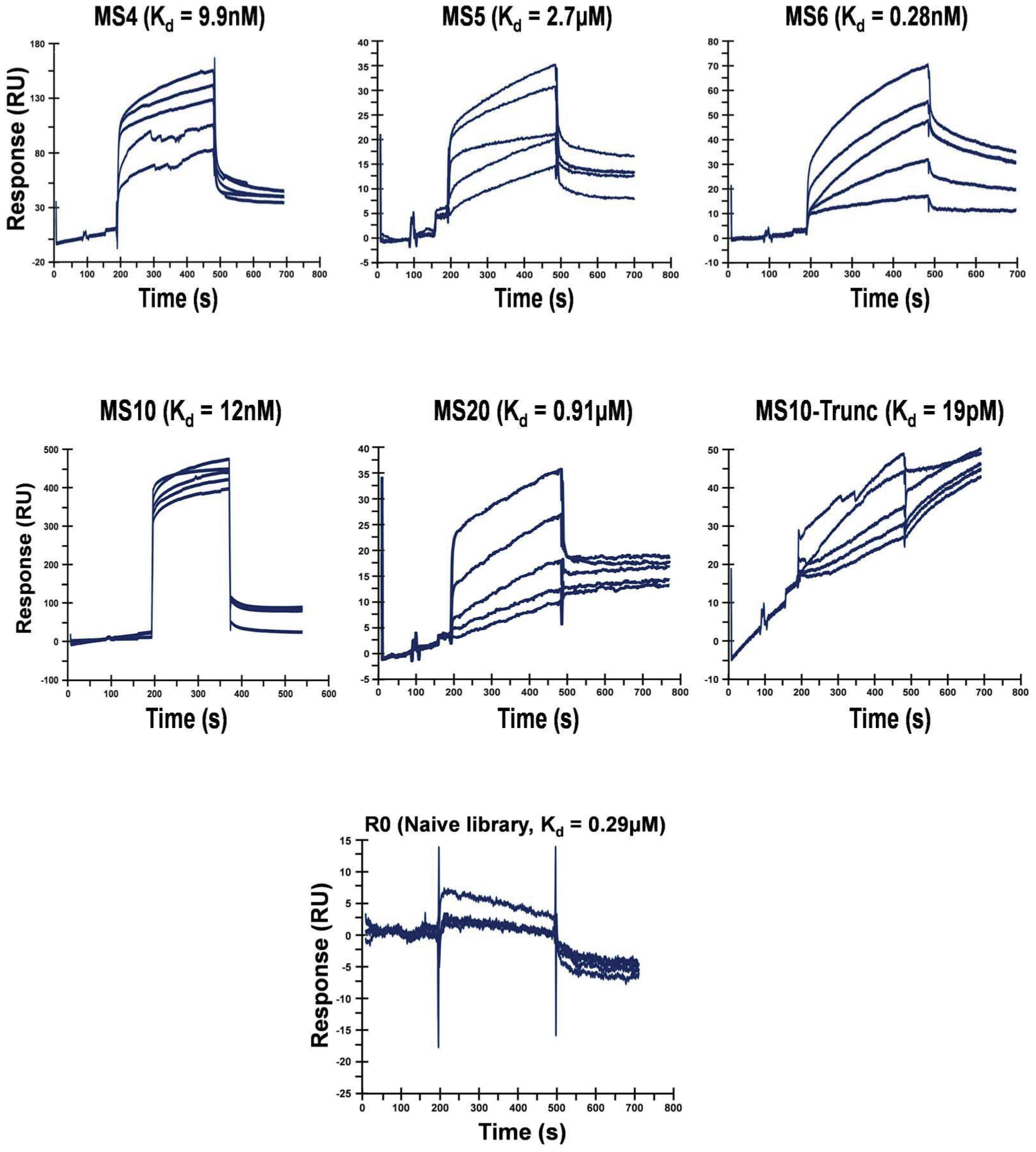

Supplementary Figure S5

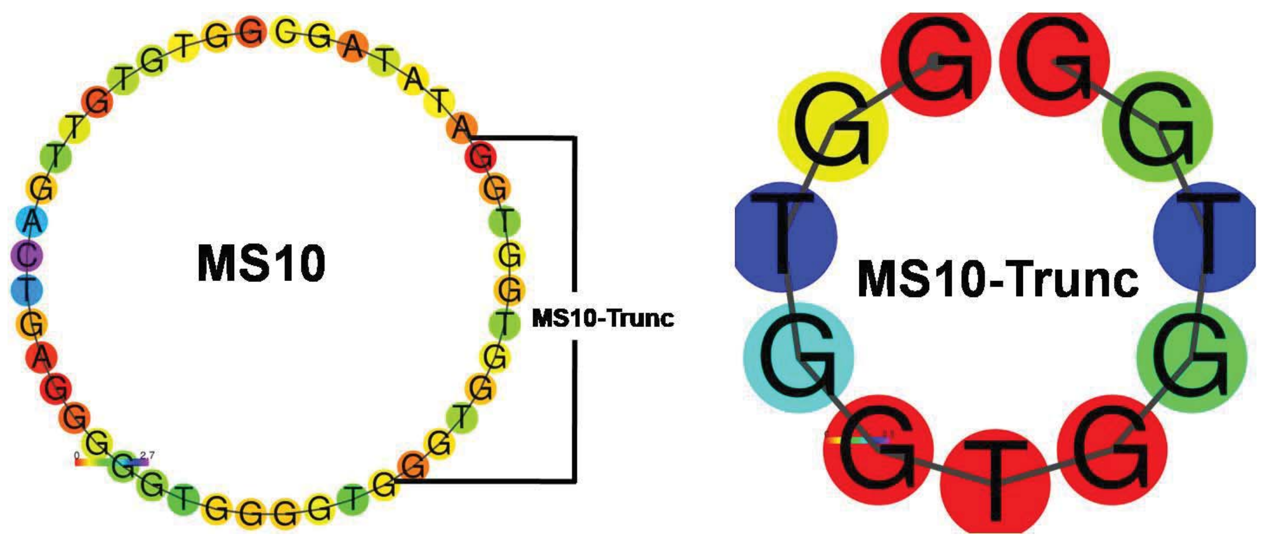

Supplementary Figure S6

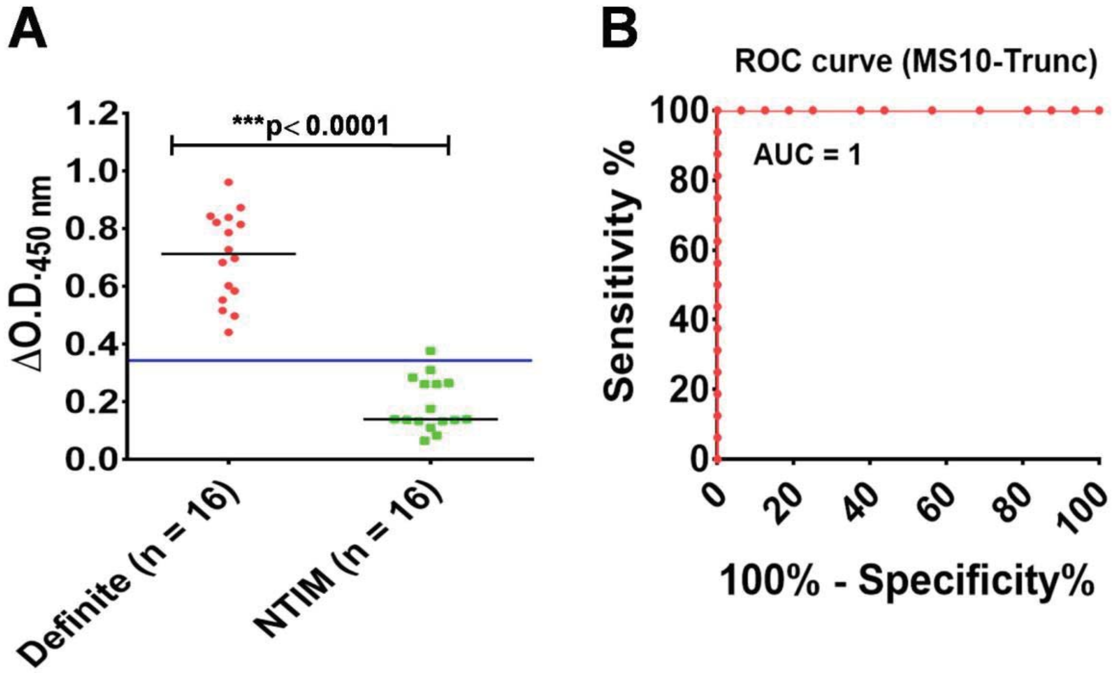

Supplementary Table S1: Sequence of aptamers subjected to QGRS mapper analysis and comparison of their G-scores with those of reported G-quadruplex (G4) forming aptamers.

S.No.	Name	Sequences*	G-Score	Length of G4 forming region (nt)	References
1.	MS4	GGACCAGGTGAGTGGATGC**GGGTGCGGGTGGGGGTGAGGG**GGGG	42	21	This study
2.	MS5	GCAGAGAGAGAGAAAGTGAGAA**GGGCGGGGGGGAGAGGGTGGGG**	40	22	
3.	MS6	GGTGGGAT**GGGGGGGGGAGGGTAGGG**TGTGCAAGTAGAGGGTC	42	18	
4.	MS10	GGTGTGTTGACTGAGGGGGTGGGGTG**GGTGGTGGTGG**ATATAGC	21	11	
5.	MS20	GGGTTGTAAGAGGGAGCT**GGGAGGGGTCGGGGGGGG**GGGTGGC	42	18	
6.	Anti HIV-1 integrase	GGGTGGGAGGAGGG	21	14	3
7.	Thrombin	GGTTGGTGTGGTTGG	20	15	4
8.	Anti-VEGF	GGGGTGGACGGGCCGGG	21	17	5

*** G4 forming regions in the various aptamers are underlined and in red.**

Supplementary Table S2: Primary nucleotide sequence of the control aptamers used in the study.

S.No.	Name	Control Aptamers
1.	C1	5’CCCCGTTGCGTCCCGTCTAGACCTAATATGATCCACGAATTATC3’
2.	C2	5’TTTTTTTTTTTTTTTTTTTTTTTTTTTTTTTTTTTTTTTTTTTTTTT3’

Supplementary Table S3: Nucleotide sequences of MS10 aptamer, its truncated and mutant derivatives. Bold nucleotides represent the truncated portion of aptamer MS10. Red highlighted base represent the site of mutation (deletion).

S.No.	Aptamer Name	Sequence
1.	MS10	GGTGTGTTGACTGAGGGGGTGGGGTG**GGTGGTGGTGG**ATATAGC
2.	MS10-Trunc	**GGTGGTGGTGG**
3.	MS10-Trunc ΔT3	**GGTGGTGGTGG**
4.	MS10-TruncΔT6	**GGTGGTGGTGG**
5.	MS10- Trunc ΔT9	**GGTGGTGGTGG**
