# Supplementary material for: Theranostic Application of a Novel G-Quadruplex-Forming DNA Aptamer Targeting Malate Synthase of Mycobacterium tuberculosis
Source: Mol Ther Nucleic Acids. 2019 Oct 4;18:661–72. doi: 10.1016/j.omtn.2019.09.026 (PMC6849348; doi:10.1016/j.omtn.2019.09.026)
Supplement: Document S2. Article plus Supplemental Information [file mmc2.pdf]

Molecular Therapy
Nucleic Acids
Original Article

Theranostic Application of a Novel G-Quadruplex-Forming DNA Aptamer Targeting Malate Synthase of Mycobacterium tuberculosis

Abhijeet Dhiman,[1,8] Chanchal Kumar,[1,10] Subodh Kumar Mishra,[2,9] Kriti Sikri,[1,9] Ishara Datta,[1] Pradeep Sharma,[3] Tej P. Singh,[3] Sagarika Haldar,[4,5] Neera Sharma,[6] Anjali Bansal,[7] Yusra Ahmad,[8] Amit Kumar,[2] Tarun Kumar Sharma,[5] and Jaya Sivaswami Tyagi[1,5]

[1]Department of Biotechnology, All India Institute of Medical Sciences, New Delhi 110029, India; [2]Discipline of Biosciences and Biomedical Engineering, Indian Institute of Technology Indore, Simrol, Indore 453552, India; [3]Department of Biophysics, All India Institute of Medical Sciences, New Delhi 110029, India; [4]Department of Experimental Medicine and Biotechnology, PGIMER, Sector 12, Chandigarh 160012, India; [5]Multidisciplinary Clinical and Translational Research Group, Translational Health Science and Technology Institute, Faridabad, Haryana 121001, India; [6]Department of Biochemistry, Dr. Ram Manohar Lohia Hospital, New Delhi 110001, India; [7]Department of Pediatrics, Dr. Ram Manohar Lohia Hospital, New Delhi 110001, India; [8]Faculty of Pharmacy, Uttarakhand Technical University, Dehradun 248007, Uttarakhand, India

The successful management of tuberculosis (TB) requires efficient diagnosis and treatment. Further, the increasing prevalence of drug-resistant TB highlights the urgent need to develop novel inhibitors against both drug-susceptible and drug-resistant forms of disease. Malate synthase (MS), an enzyme of the glyoxylate pathway, plays a vital role in mycobacterial persistence, and therefore it is considered as an attractive target for novel anti-TB drug development. Recent studies have also ascribed an adhesin function to MS and established it as a potent diagnostic biomarker. In this study, a panel of *Mycobacterium tuberculosis* (Mtb) MS-specific single-stranded DNA aptamers was identified by Systematic Evolution of Ligands by EXponential enrichment (SELEX). The best-performing G-quadruplex-forming 44-mer aptamer, MS10, was optimized post-SELEX to generate an 11-mer aptamer, MS10-Trunc. This aptamer was characterized by various biochemical, biophysical, and *in silico* techniques. Its theranostic activity toward Mtb was established using enzyme inhibition, host cell binding, and invasion assays. MS10-Trunc aptamer exhibited high affinity for MS (equilibrium dissociation constant [K_D] ~19 pM) and displayed robust inhibition of MS enzyme activity with IC_{50} of 251.1 nM and inhibitor constant (K_i) of 230 nM. This aptamer blocked mycobacterial entry into host cells by binding to surface-associated MS. In addition, we have also demonstrated its application in the detection of tuberculous meningitis (TBM) in patients with sensitivity and specificity each of >97%.

INTRODUCTION

Active tuberculosis (TB) affected more than 10.4 million people in 2017, whereas an estimated 1.7 billion individuals (about a quarter of the world's population) are estimated to harbor a latent/persistent infection with *Mycobacterium tuberculosis* (Mtb).[1] The current treatment for TB is prolonged, and drug-resistant cases are increasing,[2] which together pose a significant threat to TB control in the community.[3,4] Therefore, there is an urgent need to develop new inhibitory compounds or molecules that have the potential to work against both the drug-susceptible and resistant TB.

Metabolic pathways of central metabolism have attracted attention in recent times as a source of new targets for novel TB drug development. The glyoxylate shunt pathway in particular is considered as a prominent target pathway owing to its role in mycobacterial survival and persistence, as demonstrated in cell and animal models of Mtb infection.[5,6] This two-enzyme pathway enables bypass of the decarboxylation steps in the tricarboxylic acid cycle and conserves carbon for subsequent gluconeogenesis. Isocitratelyase, the first enzyme of this pathway, generates glyoxylate and succinate from isocitrate, and malate synthase (MS), the second enzyme, catalyzes the formation of malate using glyoxylate and acetyl-CoA (coenzyme A).[5] Notably, these enzymes appear to be absent in mammals,[5] a feature that enhances its relevance as an anti-TB drug target. In addition to its enzymatic activity, MS was shown to be expressed on the cell wall of Mtb and impart host-adhering function to bacteria, thus establishing an additional role for it as an adhesin.[7] Furthermore, MS is reported as a potential biomarker for TB infections because it is expressed in the early stage of infection and independent of HIV

Received 9 July 2019; accepted 27 September 2019;
https://doi.org/10.1016/j.omtn.2019.09.026.

[9]These authors contributed equally to this work.

[10]Present address: Department of Forensic Science, Guru Ghasidas Vishwavidyalaya, Bilaspur, Chattisgarh-495009, India.

Correspondence: Jaya S. Tyagi, Department of Biotechnology, All India Institute of Medical Sciences, New Delhi 110029, India.
E-mail: jayatyagi.aiims@gmail.com
Correspondence: Tarun K. Sharma, Multidisciplinary Clinical and Translational Research, Translational Health Science and Technology Institute, Faridabad, Haryana 121001, India.
E-mail: tarun@thsti.res.in

This is an open access article under the CC BY license (http://creativecommons.org/licenses/by/4.0/).

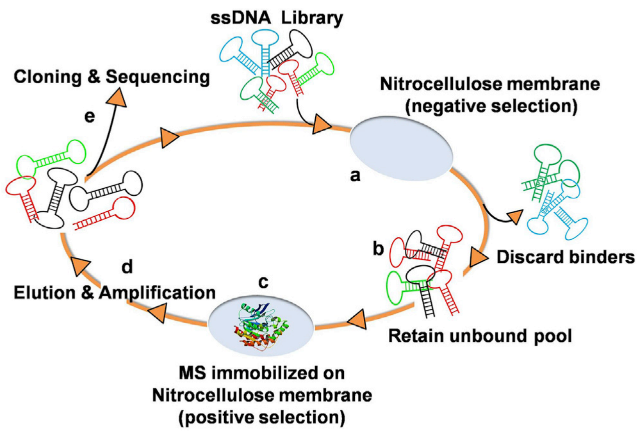

Figure 1. Systematic Enrichment of Aptamers against MS
Negative selection was performed by incubating the random ssDNA library (RDL) with nitrocellulose membrane (NCM) (step a) and then partitioning of bound and unbound sequences (step b); the unbound DNAs were collected and then incubated with immobilized MS for positive selection (step c); after washing, the bound DNAs were eluted and amplified by PCR for the next round of SELEX (step d); after enrichment, the DNA population was cloned and sequenced to identify the sequences of individual monoclonal aptamers (step e).

co-infection.[8] Thus, the presence of this antigen in biological fluid is considered as direct evidence of infection.

The crystal structures of both isocitratelyase and MS have been solved,[9,10] and insights from these structures have suggested MS as a more druggable target in comparison with isocitrate lyase.[5] A recent study demonstrated that knockdown and knockout of MS leads to Mtb clearance in the mouse model of infection, showing the essentiality of MS for the survival of Mtb during the acute and chronic phases of infection.[11] Therefore, owing to the attractiveness of MS as a drug target, inhibitors of MS have been reported, such as oxalate, phosphoenolpyruvate (PEP), bromopyruvate (BP), and glycolate, that exhibit high inhibitor constant (K_i) values ranging between 60 and 900 μM.[10] These inhibitors have limitations in terms of their use in humans; oxalate is not pharmacologically suitable due to its toxic nature, PEP is a key constituent of glycolysis and gluconeogenesis pathways in all organisms including humans, whereas BP exhibits toxicity impairing mitochondrial function.[12,13] Recently, a series of phenyl-diketo acid (PDKA) molecules having inhibitory activity against Mtb MS enzyme was also developed that is under optimization.[14,15]

A recent study from our group has established the clinical utility of polyclonal antibody to MS for the diagnosis of tuberculous meningitis (TBM), the most lethal form of tuberculosis that affects the central nervous system.[8] However, polyclonal antibody has an inherent problem of batch-to-batch variation; thus, it is difficult to scale up the assay to a diagnostic test level. To address this limitation associated with antibody, it is imperative to develop a diagnostic reagent of uniform quality. Nucleic acid aptamers have received a great deal of attention because of their enormous potential to be used as both therapeutic and diagnostic agents. By acquiring a complex 2D and 3D structure, DNA aptamers often recognize their cognate target with unmatched affinity and specificity.[16,17]

Here we report the development of a G-quadruplex-(G4)-forming aptamer MS10-Trunc against MS of Mtb that can serve as a novel theranostic molecule for the management of TB.

RESULTS

In the current study, high-affinity MS-specific single-stranded DNA (ssDNA) aptamers were identified from an 80-mer random DNA library (RDL) using SELEX-based screening. The best-performing aptamer candidate, MS10, was further optimized post-SELEX to generate MS10-Trunc.The theranostic potential of MS10-Trunc was established by its inhibitory activity against MS enzyme, interference of Mtb binding and invasion into host cells, and ability to detect MS in cerebrospinal fluid (CSF) specimens for the diagnosis of TBM.

Aptamer Selection and Screening for MS Aptamers

Mtb MS was expressed in *E. coli* BL21(DE3)pLysS, purified through affinity chromatography (Ni-NTA), and used as a target to generate MS-specific aptamers by the process of SELEX (Figure 1). The SELEX process included a step of negative selection to exclude nitrocellulose membrane (NCM) binders (step a), followed by partitioning of unbound sequences (step b) and a positive selection step to obtain binders to MS coated on NCM (step c). The bound DNA was eluted and amplified (step d) followed by the generation of ssDNA for the next round of SELEX. After 10 iterative rounds of SELEX, the archived aptamer populations from rounds 2, 4, and 6–10 were assessed for binding to MS using Aptamer Linked Immobilized Sorbent Assay(ALISA). The enrichment of MS-binding aptamers was evident over consecutive rounds of SELEX (Figure 2A). However, the aptamer population of rounds 8–10 evinced the highest binding over the random DNA library (RDL). Interestingly, the round 9 aptamer population showed higher binding to MS compared with the round 10 aptamer pool (Figure 2A). The reason for this could be the improper partitioning between specific and non-specific MS binders during the 10th round of selection process or a decrease in the aptamer pool complexity, or a combination of both.[18] The aptamers of rounds R8, R9, and R10 were individually cloned in the TA cloning vector pTZ57R/T, and clones were randomly picked and sequenced (Figure 1, step e). Based on the sequencing results, representative aptamer candidates from each round were subjected to CLUSTAL W and BioEdit analysis to identify the primary sequence homology and nucleotide richness (Figure S1). Based on the nucleotide richness, all of the 22 aptamer candidates were categorized into two groups, G-rich and non-G-rich (Figure S1). Interestingly, ~50% of aptamer candidates were G-rich with high G+C content (Figures S2A and S2B), suggesting the preferential binding of MS to G-rich DNA aptamers. These 22 monoclonal aptamer candidates were first screened for their binding to MS by ALISA (Figure 2B) and 5 aptamers, namely, MS4, MS5, MS6, MS10, and MS20, were selected for further study using an arbitrary cutoff of $\Delta OD_{450nm} \geq 0.6$. Among them, MS4 and MS20 were derived from the R8 pool, and MS5 and MS6 belonged to the R9 pool, whereas MS10 was obtained from the R10 aptamer population.

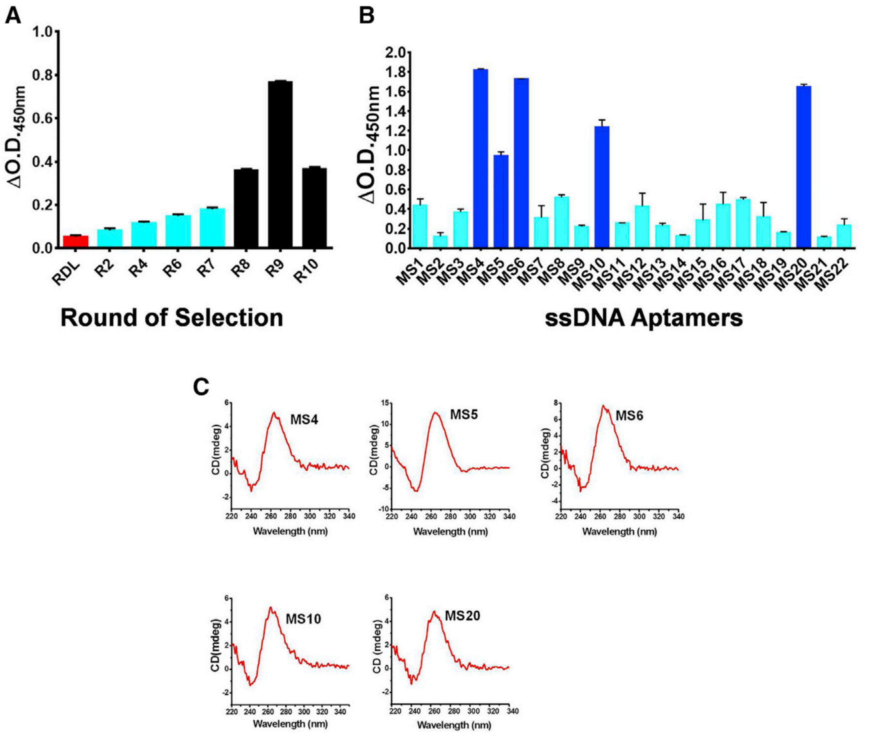

Figure 2. Showing Binding of Aptamer Pool and Monoclonal Aptamer Candidate to MS and CD-based Structural Evaluation of Aptamer Structure

(A) Relative binding of various aptamer pools (rounds 2, 4, and 6–10 and RDL) to target MS as determined by aptamer-linked immobilized sorbent assay (ALISA). (B) Relative binding of various monoclonal aptamers with MS. The aptamers highlighted in blue were selected for further characterization based on an arbitrary cutoff $OD_{450nm} \geq 0.6$. (C) CD spectra of MS aptamer candidates.

In Silico Secondary Structure Prediction of Aptamers

To gain structural insights into aptamers, we predicted their secondary structure by a web-based tool, RNA fold, using DNA parameters (http://rna.tbi.univie.ac.at/cgi-bin/RNAWebSuite/RNAfold.cgi). MS4 and MS20 acquire a typical stem-loop structure, whereas MS5, MS6, and MS10 form only a loop-like structure (Figure S3). The selected aptamer candidates belong to the G-rich category that usually has a propensity to form G4 structures. Analysis of the sequences using an *in silico* tool QGRS Mapper (http://bioinformatics.ramapo.edu/QGRS/index.php) and G4IPDB (http://bsbe.iiti.ac.in/bsbe/ipdb/index.php) predicted that all the queried aptamers, namely, MS4, MS5, MS6, MS10, and MS20, form G4 structure and show scores comparable or higher than the known G4-forming aptamer sequences (anti-thrombin, anti-HIV integrase, and vascular endothelial growth factor [VEGF] aptamers)[19–21] (Table S1).

Further, to validate *in silico* the predictions, we undertook circular dichroism (CD) spectroscopy analysis, which revealed that all of the aptamers form G4 structures. Each aptamer shows a negative peak near 242 nm and a positive peak close to 265 nm, which typically signify a parallel G4 structure formation by all of these aptamers[22–24] (Figure 2C).

Determination of Dissociation Constant of Aptamers by Surface Plasmon Resonance

Purified MS was amine-coupled onto a CM5 chip through 1-ethyl-3-(3-dimethylaminopropyl) carbodiimide and *N*-hydroxysuccinimide (EDC-NHS) chemistry, followed by injection of the individual aptamers, to obtain the equilibrium dissociation constant (K_D) values for each aptamer. All of the aptamers exhibited tight binding with K_D in low micromolar to nanomolar range; the aptamers were ranked in terms of decreasing affinity (K_D values) as MS6 (0.28 nM) > MS4 (9.9 nM) > MS10 (12 nM) > MS20 (0.91 μM) > MS5 (2.7 μM) (Table 1; Figure S4).

Inhibition of MS Enzyme Activity by Aptamers

Purified MS protein was pre-incubated with selected aptamers (namely, MS4, MS5, MS6, MS10, and MS20) for 30 min followed by the addition of glyoxylate and acetyl-CoA to complete the reaction. The inhibition of MS enzyme activity by various aptamers was monitored by estimating free CoA as described in Materials and Methods.[10] Based on the extent of inhibition of MS activity, the aptamers were ranked as MS10 > MS5 > MS20 > MS4 > MS6. MS10 was determined to be the most potent inhibitor; it evinced inhibitory activity of ∼91% against purified MS protein (Figure 3A). A control aptamer of the same length (C1; Table S2) showed negligible inhibition of MS activity and suggested that MS10 aptamer-mediated inhibition was sequence specific.

Among all the tested aptamers, MS10 aptamer exhibited maximum inhibitory activity against MS. Therefore, MS10 aptamer was taken up for further studies.

Post-SELEX Optimization of MS10 Aptamer

In the literature, numerous G4-forming aptamers are reported as potent inhibitors of biological activity.[25] MS10 was a G-rich aptamer and to establish its structure-activity relationship (SAR), its G4-forming sequence that was identified through QGRS Mapper (Figure S5) was synthesized and designated as MS10-Trunc (5′-GG TGGTGGTGG-3′) and assessed for its ability to bind MS using ALISA. The 11-mer MS10-Trunc displayed three GGT repeats and exhibited similar binding to MS compared with its 44-mer parent aptamer MS10 (Figure 3B), thus representing the smallest functional unit of the parent aptamer. To establish the role of each "T" nucleotide present in MS10-Trunc, the T nucleotides were mutated by deleting one T nucleotide at a time (Table S3), and the binding of individual mutant aptamers (ΔT3, ΔT6, and ΔT9) to MS was assessed

Table 1. Surface Plasmon Response (SPR) Binding Kinetics Data of Aptamers

Aptamer	K_{on} (1/ms)	K_{off} (1/s)	K_A (1/M)	K_D (M)
MS4	2.4×10^5	2.4×10^{-3}	1×10^8	9.9×10^{-9}
MS5	3.7	1×10^{-5}	3.7×10^5	2.7×10^{-6}
MS6	3.6×10^4	1×10^{-5}	3.6×10^9	2.8×10^{-10}
MS10	2.8×10^5	3.2×10^{-3}	8.7×10^7	1.2×10^{-8}
MS20	11	1×10^{-5}	1.1×10^6	9.1×10^{-7}
MS10-Trunc	5.2×10^5	1.6×10^{-5}	5.2×10^{10}	1.9×10^{-11}
R0 (naive library)	35	1×10^{-5}	3.4×10^6	2.9×10^{-7}

K_A, association constant.

by ALISA. In terms of aptamer binding to MS, the deletion of T3 or T9 was well tolerated, whereas the deletion of T6 led to ~28% reduction in aptamer binding compared with MS10-Trunc (Figure 3B), indicating a crucial role for T6 in maintaining the stable G4 aptamer structure. To further evaluate the impact of truncation on aptamer binding, we determined the K_D of MS10-Trunc using surface plasmon resonance (SPR) to be 19 pM (Figure S4), indicating an ~631-fold improvement in affinity in comparison with the parent aptamer, MS10 (K_D = 12 nM; Table 1).

Determination of Inhibition Kinetics of MS10 and Its Truncated Derivative, MS10-Trunc

The 11-mer MS10-Trunc aptamer exhibited a similar extent of inhibitory activity (~94%) as compared with the parent aptamer MS10 (~91%; Figure 3C). A comparative inhibition study was performed for MS10 and MS10-Trunc, along with the known MS inhibitors, phosphoenolpyruvate (PEP) and bromopyruvate (BP).[10] The IC_{50} values of MS10 and MS10-Trunc were determined to be 275.6 and 251.1 nM, whereas for BP and PEP they were 114.3 and 374.1 μM, respectively (Figure 4A), indicating that MS10-Trunc was more potent than its parent aptamer, MS10, as well as the known inhibitors, BP and PEP. Although the IC_{50} value of an inhibitor is an indicator of its functional strength (such as inhibiting enzyme activity), the K_i value (the equilibrium dissociation constant of the inhibitor) is more reflective of its binding affinity for the target. We further determined the K_i value for MS using linear replots of Lineweaver-Burk slopes using a range of aptamer and substrate concentrations. MS10 and MS10-Trunc showed K_i of 270 and 230 nM, respectively (Figures 4B and 4C), which further supports their robust inhibitory potential. PEP and BP are reported to exhibit K_i values of ~200 and ~60 μM, respectively.[10] The parent aptamer exhibited ~740- and ~220-fold more potency than PEP and BP, whereas MS10-Trunc displayed ~870- and ~260-fold more potency than PEP and BP.

We further determined the inhibition mechanism of the MS10 and MS10-Trunc to evaluate the mode of aptamer binding to MS that leads to such robust inhibition. There was no significant change in inhibition of enzyme activity when the order of addition of aptamer or substrate to MS protein was reversed. Both MS10 and MS10-Trunc

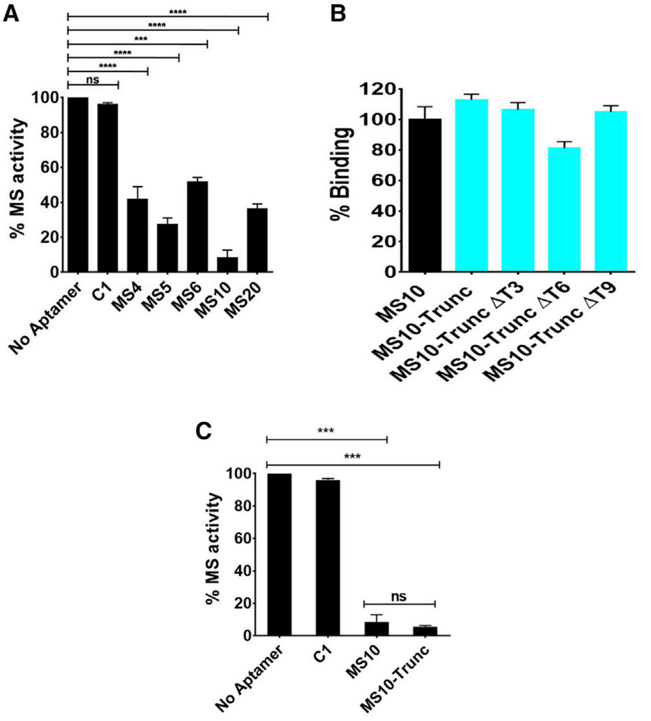

Figure 3. Showing Inhibitory Potential of Aptamer Candidates for MS
(A) Effect of aptamers on the catalytic activity of Mtb MS at a fixed aptamer concentration (2 μM). (B) Binding ability of truncated and different mutant versions of MS10 aptamer to target MS. The binding of individual mutant aptamers to MS was compared with MS10-Trunc binding to MS using ALISA. Individual base deletions are represented as cyan bars in the graph. (C) Effect of MS10 and MS10-Trunc aptamers on catalytic activity of Mtb-MS. C1 is the control aptamer (sequence is given in Table S2, used in A and C). ***p < 0.0001 was calculated using one-way ANOVA).

inhibit MS by mixed inhibition mechanism, indicating that they can inhibit catalysis regardless of whether the substrate is bound to the enzyme (Figures 4B and 4C).

Inhibition of *Mycobacterium tuberculosis* Invasion in Cells

Because MS is also expressed on the cell surface of Mtb and exhibits adhesin activity that contributes to bacterial invasion into host cells,[7] we assessed the interaction of MS10-Trunc with cell surface-associated MS. Flow cytometry analysis (Figure 5A) clearly demonstrated preferential binding of MS10-Trunc aptamer with Mtb as compared with the control oligo C2, indicating that the former aptamer interacted with cell surface-associated MS. We next assessed the ability of MS10-Trunc to modulate invasion/infection of Mtb H37Rv into human THP-1 monocytic cells. An ~40% decrease in Mtb infectivity was observed in the presence of MS10-Trunc (Figure 5B, ***$p < 0.0001$), and no such inhibition was observed with 44-mer control oligos (C1 and C2), suggesting that inhibition is aptamer specific and Mtb utilizes a MS-dependent mechanism, at least in part, for host cell entry. Taken together, these findings demonstrate the potential application of aptamer-based inhibitors as a TB therapeutic agent.

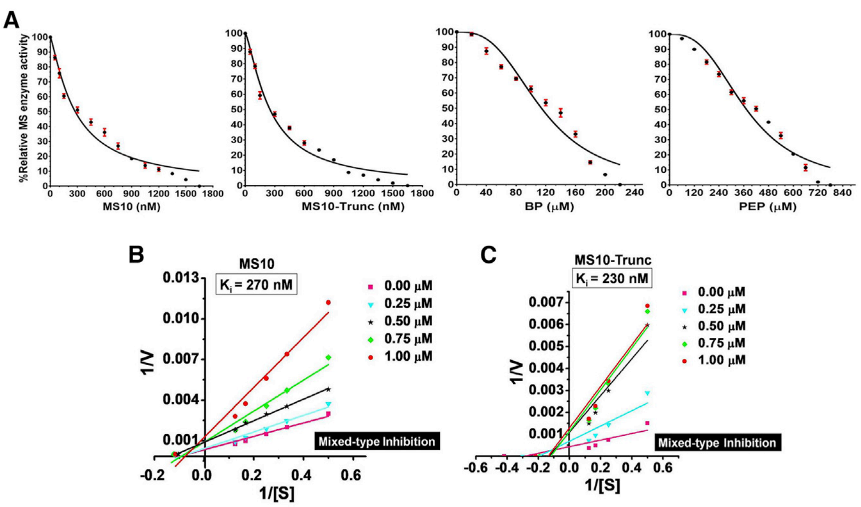

Figure 4. Showing Relative IC_{50} of Aptamer and Known MS Inhibitors and Ki of MS10 and MS10-Trunc

(A) Determination of IC50 of MS10, MS10-Trunc aptamers and known MS inhibitors (BP and PEP). The relative activity of the Mtb-MS as a function of the various concentrations of inhibitory aptamers MS10 and its truncated form MS10-Trunc or known MS inhibitors (BP and PEP). (B and C) K_i value and inhibition mechanism of aptamer MS-10 (B) and its derivative MS10-Trunc (C) were determined using double-reciprocal Lineweaver-Burk plot.

Diagnostic Application of MS10-Trunc

The diagnostic potential of MS10-Trunc aptamer to detect MS in CSF samples was assessed next in 86 archived CSF specimens belonging to TBM and disease control categories. A detailed description about "classification of CSF samples" is given in Supplemental Materials and Methods. To determine diagnostic accuracy of MS10-Trunc aptamer, we constructed a receiver operating characteristics (ROC) curve first using ΔOD_{450nm} values obtained with CSF from definite TB (true positive) and Non-tuberculous Infectious Meningitis (NTIM) group (true negative) samples, and we constructed a scatter-plot for the two sample categories. The area under the curve was 1.0, which indicated the robustness of the assay (Figures S6A and S6B). The NTIM category was composed of cases of pyogenic bacterial meningitis that included two culture-confirmed cases of *E. coli* (n = 1) and *Acinetobacter* sp. (n = 1). The remaining 14 cases were diagnosed on the basis of response to appropriate antibiotics, clinical presentation, and symptoms. Based on the cutoff derived from the ROC curve (0.3444), the performance of MS10-Trunc aptamer ALISA was evaluated (Figure 6). The aptamer MS10-Trunc yielded a very high sensitivity and specificity (>97%) with a highly significant discrimination (***$p < 0.0001$) between CSF from TBM and non-TBM subjects. This is a highly encouraging finding and requires substantiation in a larger study on geographically distinct populations so that this lead can be translated into a commercially viable diagnostic assay.

Structural Insights into MS10-Trunc

Secondary structure analysis of MS10-Trunc, the most potent inhibitor among the molecules assessed in the study, was predicted to have only a loop configuration, albeit a smaller one, as compared with MS10, its parent aptamer, suggesting that the loop is sufficient for aptamer interaction with MS (Figure S5). A recent report also suggests that the loop region is sufficient for interaction of aptamer to human erythropoietin α with its cognate target.[26] CD spectra analysis revealed that MS10-Trunc aptamer, like its parent aptamer (Figure 2C), exhibited a negative peak at 242 nm and a positive peak at 265 nm (Figure 7A), which is typical of parallel-type G4 structure.[22,23] This G4 structure was dynamic and underwent a structural change in the presence of MS in an aptamer concentration-dependent manner, as seen by changes in ellipticity (Figure 7A). The formation of G4 structure of MS10-Trunc aptamer was confirmed by ^{1}H-NMR analysis. The formation of a G4 structure by MS10-Trunc aptamer was confirmed by ^{1}H-NMR spectrum analysis; the aptamer exhibited a set of merged imino proton resonances at 10–12 ppm with broad line widths, clearly demonstrating the formation of a predominant G4 fold[27] (Figure 7B). The NMR melting temperature experiment further confirms thermostability of the DNA aptamer and shows the existence of imino signals at the higher temperature (up to 65°C). These NMR findings are consistent with *in silico* prediction of G4 structure by QGRS Mapper and the findings of CD spectral analysis.

Molecular Modeling and *In Silico* Docking Studies

Toward identifying the binding location of MS10-Trunc on MS in accordance to structural insights obtained from CD and NMR analysis, we generated a parallel inter-stranded G4 structure model of MS10-Trunc aptamer (Figure 8A). This energetically refined model contains four chains (O, P, Q, and R displayed in different color code) of identical length (11-mer), and it was selected as the input ligand for docking studies using the crystal structure of Mtb MS (PDB: 2GQ3; Figure 8). The analysis of the docked structure predicted interactions of MS10-Trunc aptamer with Thr528, Lys484, Thr482, and Gln486 residues of MS protein (Figure 9). The G10 nucleotide of R chain forms two hydrogen bonds by interacting with Thr482 and Gln486 residues of MS, G5 nucleotide of Q chain forms one hydrogen bond with Thr528, and G7 of O chain forms one hydrogen bond with Lys484. All of these residues map in the TIM barrel domain of MS, which forms the active site of MS at the interface of TIM barrel residues (115–134 and 266–557), residues of domain II (591–727), and residues of a loop region (616–633).[10]

DISCUSSION

There is a need for versatile reagents with theranostic activity that can both detect and treat tuberculosis. The selection of a unique target that can meet both diagnostic and theranostic criteria underlies the successful design of a useful theranostic agent. MS is the second and terminal enzyme of the glyoxylate cycle that is essential for sustaining a persistent TB infection[10] of both drug-susceptible and drug-resistant forms of TB. This enzyme is absent in humans, which makes

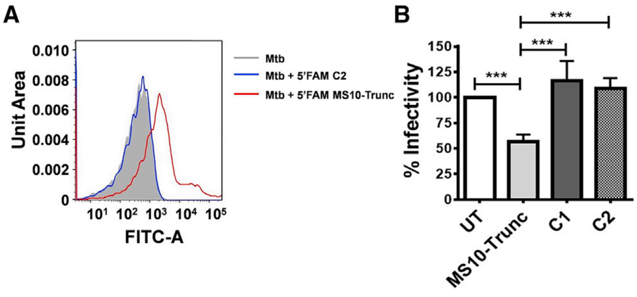

Figure 5. Inhibition of Mtb Infection with MS10-Trunc in the THP-1 Cell Infection Model

(A) Binding of MS aptamer to Mtb H37Rv determined by flow cytometry using 5′-FAM-labeled aptamers. C2 was used as control aptamer (sequence was given in Table S2). (B) Aptamer-treated Mtb was infected in THP-1 monocytes and intracellular bacteria plated on MB agar post-infection for CFU determination. The graph shows the percentage infectivity calculated with respect to the untreated (UT, no aptamer treatment) control. ***$p < 0.0001$ (calculated using one-way ANOVA). C1 and C2 were used as control aptamer (sequence was given in Table S2, used in B). The difference in infectivity between UT, C1, and C2 was found to be non-significant.

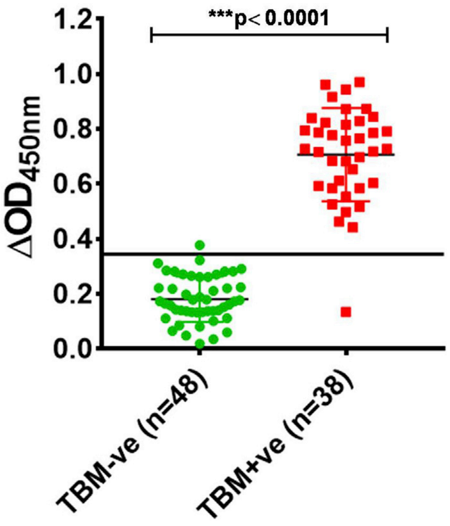

Figure 6. MS10-Trunc aptamer-based detection of MS antigen by ALISA in archived CSF samples categorized as TBM patients (n = 38) and non-TBM patients (n = 48).

MS an attractive TB drug target.[15,28] Furthermore, in recent years its role as a biomarker for detecting active TB was also established.[7,8] Thus, taking together these attributes, MS can be considered as a potent and novel theranostic target for the management of TB.

In recent years, nucleic acid aptamers have gained significant attention from the scientific community as promising inhibitors and drug candidates because of their rapid synthesis, high stability, non-toxic nature, ease in optimization and functionalization, and ability to control pharmacokinetic and pharmacodynamic parameters.[29–31] We have developed a panel of parallel G4 structure-forming ssDNA aptamers that showed high-affinity binding and robust inhibition of MS catalytic activity using SELEX technology. An 11-mer G4 structure-forming aptamer, MS10-Trunc, having high-performance characteristics, was developed by post-SELEX optimization of its parent aptamer, MS10. Post-SELEX optimization led to ~630-fold improvement in K_D of the 11-mer for MS over its full-length 44-mer counterpart. This finding is consistent with previously published reports where an improvement in binding affinity is reported on aptamer truncation.[32] Further, MS10-Trunc demonstrated a high inhibitory potential, with low IC_{50} (230 nM) that was comparable with that of its parent aptamer MS10 (270 nM). IC_{50} data unequivocally demonstrated that the inhibitory activity of MS10-Trunc is considerably superior (~1,000 fold) to that of the known MS inhibitors, BP and PEP,[10] and comparable with that of recently reported PDKA inhibitors that had IC_{50} values in the range of 0.01–0.6 μM.[14] There are reports that G4-forming structures allow aptamers to interact strongly with their cognate targets, such as anti-thrombin aptamer,[19] nucleolin-targeting aptamer,[33] and anti-VEGF aptamer.[31] More recently, G4-forming inhibitory DNA aptamers against Mtb polyphosphate kinase-2 were also reported.[4] A truncated aptamer with similar length (11-mer) is previously reported by Nadal et al.[34] for detecting β-conglutin, an allergen. The high-affinity binding properties of MS10-Trunc to MS can be ascribed to its ability to form an 11-mer G4 structure (MS10-Trunc) that was confirmed by CD spectroscopy analysis and ^{1}H-NMR studies.

In silico modeling and docking studies have provided useful insights into the molecular interactions between MS10-Trunc aptamer and its cognate target, MS, that may contribute to its theranostic activity. *In silico*-generated parallel G4 structure of MS10-Trunc was predicted to have a four-stranded structure containing R, O, Q, and P chains. This observation is in agreement with the previous reports where a 6-mer anti-HIV DNA molecule forms a similar parallel G4 structure involving four chains.[35] G-nucleotides mapped to the R, O, and Q chains of G4 structure of MS10-Trunc were predicted to have H-bonding interactions with four amino acid residues (Thr482, Thr528, Lys484, and Gln486) that are located in the TIM barrel domain of MS. Because the TIM barrel domain forms the active site interface by interacting with the residues of domain II and loop region (616–663), binding of MS10-Trunc aptamer to residues of the TIM barrel domain may possibly lead to a conformational change in/near the active site of MS that adversely influences catalytic function of MS and results in an inhibition of enzymatic activity of MS in the presence of MS10-Trunc. It is also established that MS acts as an adhesin and is known to play a vital role in bacterial invasion into host cells.[7] Our findings clearly demonstrate that MS10-Trunc aptamer blocks bacterial invasion by ~40%. In addition to MS, Mtb is also known to use other adhesins, including but not limited to HupB, PstS-1, and LpqH (19-kDa antigen),[36] during host cell invasion. A previous study has also reported aptamer-mediated inhibition of Mtb entry into murine macrophages, although the identity of the aptamer target was not known.[37] Therefore, to achieve more robust inhibition of bacterial invasion, a combinatorial approach (a cocktail of aptamers targeting various adhesins of Mtb) may possibly be used.

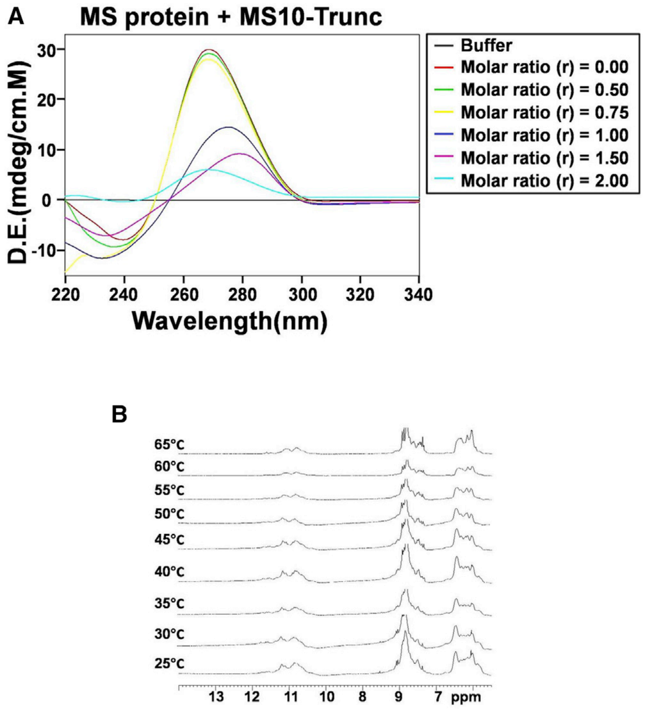

Figure 7. Structural Evaluation of MS10-Trunc

(A) Circular dichroism titration spectrum for MS10-Trunc aptamer (red) and aptamer in the presence of increasing concentration of MS up to protein (P)/aptamer (A) ratio = 2.0. (B) Temperature-dependent ^{1}H-NMR spectra for MS10-Trunc showing imino and base region.

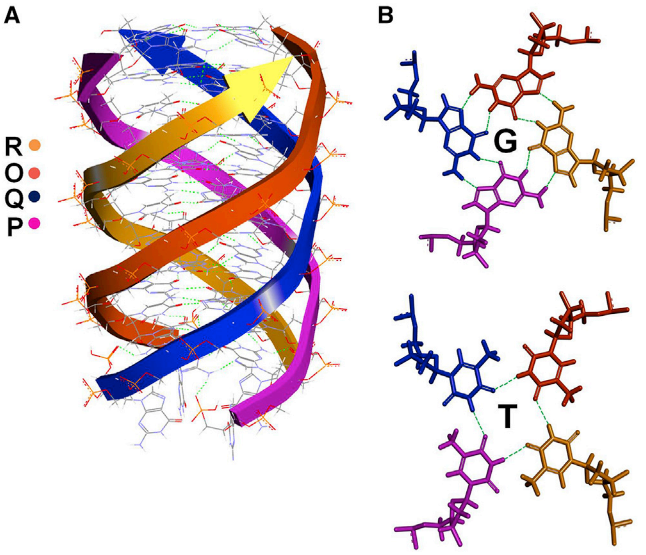

Figure 8. Model Structure of MS10-Trunc

(A and B) A model of G4 structure formed by MS10-Trunc aptamer (A) and hydrogen bonds in each G and T quartet of G4 structure (B).

This study offers proof of concept for a TB theranostic reagent that is directed against MS. Our findings indicate that MS10-Trunc aptamer has the potential to target Mtb infection. However, the stability and delivery of aptamers into mycobacteria pose a challenge for their therapeutic application. Although G4-based aptamers are relatively more stable in comparison with their non-G4 counterparts, their stability may be further improved by chemical modification strategies.[38,39] Efforts are ongoing in our laboratory to suitably modify the aptamer using various functionalization and conjugation strategies to achieve high stability and efficient delivery of MS10-Trunc aptamer into Mtb.

The future challenges relating to aptamer delivery in animal models or humans can possibly be addressed by administration of aptamer in the form of an inhaled aerosol mist or by injecting the aptamer into the blood. The delivery of aptamer through the inhalation route might protect the truncated aptamer from the exonucleases in serum, whereas delivery of aptamer as injectables would require suitable modification such as blockage of the 3′ end with polyethylene Glycol (PEG), modification of the backbone, introduction of modified bases, and so on to avoid enzymatic breakdown and rapid clearance by the kidneys and liver.

The performance of the aptamer-based TBM diagnostic test developed in the current study is superior to various previously reported diagnostic modalities (Table 2). MS10-Trunc aptamer has shown a very high diagnostic accuracy (>97% sensitivity and specificity) with an ability to efficiently discriminate between TBM and non-TBM subjects (***$p < 0.0001$). This aptamer, being a synthetic DNA molecule, will evince minimal/no batch-to-batch variation and can easily be adapted onto various biosensing platforms for developing a rapid point-of-care test for TBM and possibly other forms of TB.

In conclusion, we have identified a panel of G4-forming ssDNA aptamers that represents a novel class of theranostic molecules against Mtb MS. The theranostic potential of the best-performing and optimized G4-forming aptamer, MS10-Trunc, was established through binding studies using ALISA and SPR, enzyme inhibition assay, binding and inhibition of Mtb invasion into host cells, and demonstrating its diagnostic utility in TBM. We anticipate that MS10-Trunc holds promise for the development of a novel MS-targeted theranostics approach against Mtb and may complement the existing diagnostic and therapeutic modalities in the near future.

MATERIALS AND METHODS

Reagents and Samples

All oligonucleotides and other primers utilized in the study were purchased from Integrated DNA Technologies (IDT, USA). Other routine reagents and chemicals were purchased from Sigma-Aldrich, USA. Bacterial expression plasmids of MS (GlcB), MPT51, MPT64, and CFP-10; purified proteins Ag85C, HspX, GroES, LAM, and ESAT-6; and culture filtrate proteins (CFPs) were provided by BEI Resources and NIAID, NIH (please refer to Acknowledgments for details).

Purification of MS

Plasmid pMRLB.8 encoding C-terminal hexa-histidine fusion of Mtb MS (Rv1837c) was used to overexpress recombinant MS protein in

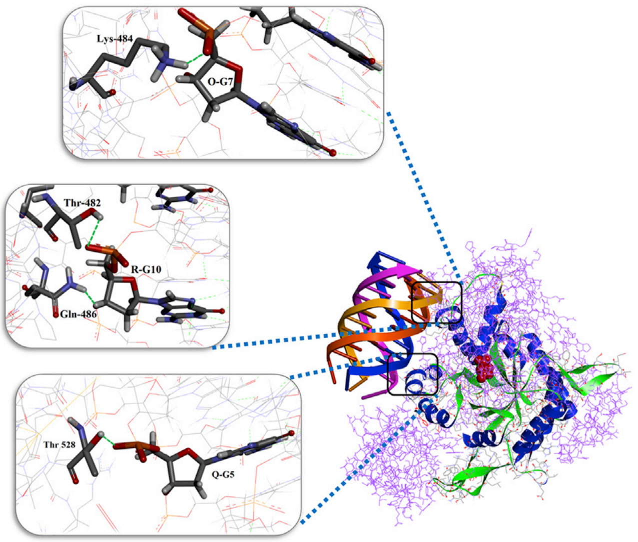

Figure 9. The Interaction of PEP (Red) and MS10-Trunc (Multicolor) with MS
The interactions between residues in MS protein and bases in aptamer are shown in cartoon representation, and the regions in the box are shown enlarged in ball-and-stick model. The interactions are shown as blue dashes.

E. coli BL21(DE3)pLysS. Bacterial cultures were grown in 2× yeast tryptone (YT) medium at 37°C to an OD_{595} of ~0.5 followed by an induction with 1 mM isopropyl-1-thio-β-D-galactopyranoside (IPTG) for 5 h at 25°C. MS was purified from the induced culture by Ni-NTA affinity chromatography as described previously.[8]

Selection of DNA Aptamer against Mtb MS Antigen Using SELEX

A synthetic 80-mer single-stranded (ss) random DNA library (RDL) containing a random region of 44 nt flanked by fixed primer binding sequences of 18-mer each that was used in our previous study[40] was employed to screen MS-specific aptamers. The fixed primer binding sequences allow DNA amplification by PCR using DNA Random Forward primer (DRF) (forward 5′-GTC TTG ACT AGT TAC GCC-3′) and DNA Random Reverse primer (DRR) (reverse 5′-GAG GCG CCA ACT GAA TGA-3′) primers. The subtractive nitrocellulose membrane (NCM)-based SELEX approach was used as described. For round 1, 10 μg of MS was blotted onto the NCM and allowed to dry at room temperature (RT). Meanwhile the RDL (1,500 pmol) was heated at 92°C followed by snap chilling on ice and finally brought to RT. The denatured library was first incubated with NCM in a step of negative selection. The unbound sequence pool was then incubated with pre-dried NCM blotted with MS in SELEX screening buffer (10 mM Tris [pH 7.5] supplemented with 10 mM $MgCl_2$, 50 mM KCl, 25 mM NaCl, 0.05% Tween 20 [v/v], 0.1 μg/μL yeast tRNA, and 0.1 μg/μL BSA). The unbound ssDNA was washed away with selection buffer (SB; 10 mM Tris [pH 7.5], 10 mM $MgCl_2$, 50 mM KCl, 25 mM NaCl) supplemented with 0.5% Tween 20. The bound DNA was eluted using nuclease-free water, followed by heating at 92°C for 5 min. The eluted DNA binder population was used as a template for PCR amplification with the forward and reverse primers for an optimized number of cycles of thermal cycling: 94°C for 30 s, 55°C for 30 s, and 72°C for 30 s with a final extension at 72°C for 5 min. PCR cycle numbers were optimized in order to avoid formation of any undesired high-molecular-weight amplicon. Subsequently, ssDNA was prepared using alkaline treatment as described previously[40,41] and used in the next round of SELEX. The rigorousness of selection was intensified in every successive round of SELEX by increasing the strength of Tween 20 during washing and by decreasing the interaction time with the target during selection. In order to minimize cross-reactivity, counter-selection was employed by incubating the binder population with other Mtb proteins, namely, purified HspX, CFP-10, ESAT-6, Ag85C, LAM, MPT51, GroES, and culture filtrate proteins (CFPs). After 10 rounds of selection, the high-affinity pools were cloned in the pTZ57R/T vector system (InsTAClone PCR Cloning Kit; Thermo Scientific) and transformed in *E. coli* DH5α. The obtained colonies were randomly picked and subjected to DNA sequencing.

Monitoring the Rounds of SELEX

After 10 iterative rounds of SELEX, the archived aptamer populations from rounds R2, R4, R6, R7, R8, R9, and R10 were amplified through PCR using 5′ biotinylated forward primer and rA-containing reverse primer, followed by strand separation using denaturing urea-PAGE as described previously.[40] These ssDNA pools of different rounds were checked for their binding to MS by aptamer-linked immobilized sorbent assay (ALISA). For this, 500 ng of purified MS protein was coated onto a Nunc MaxiSorp 96-well plate (Thermo Scientific) overnight at 4°C followed by blocking with 5% BSA at RT. The biotinylated aptamers pool of the archived population from each round was heated at 92°C for 10 min, cooled on ice, and brought to RT. A total of 100 ng of biotinylated aptamer pool was then added to individual wells pre-coated with MS. Streptavidin-Peroxidase Polymer Ultrasensitive (Sigma) was then added at 1:2,000 dilution followed by washing with SB to remove unbound streptavidin-horseradish peroxidase (HRP) conjugate, and finally TMB (3, 3′, 5, 5′ tetramethylbenzidine) (BD OptEIA) was added. The reaction was stopped by 5% sulfuric acid followed by absorbance measurement at 450 nm.

Screening of Aptamers via Aptamer-Linked Immobilized Sorbent Assay (ALISA)

In brief, 500 ng of MS was coated in 100 μL (0.1 mol/L) of sodium bicarbonate buffer (pH 9.6) on a 96-well plate and incubated at 4°C overnight. The wells were blocked with 5% BSA solution for 2 h at RT. The rest of the ALISA procedure was followed as described previously.[40]

CD Spectra Analysis

The CD spectra was obtained on Jasco J-815 Spectropolarimeter (Jasco Hachioji, Tokyo, Japan) equipped with Peltier junction temperature controller with constant supply of nitrogen gas just to avoid water condensation around the cuvette. The path length of the used cuvette was 0.2 cm, whereas spectral interval of each scan was set as 0.1 nm and the scanning rate was fixed at 20 nm/min. A blank spectrum was recorded with SB only and subtracted from the CD

Table 2. Performance of Various Tests in Diagnosis of TBM

Diagnostic Assays for TBM Diagnosis	Sensitivity (%)	Specificity (%)	Comments	References
TB culture	27	100	poor sensitivity, long turnaround time	47
Smear microscopy	10–20	100	poor sensitivity	48
DNA-Based Tests				
Xpert MTB/RIF assay	55.1	94.8	moderate sensitivity, but an excellent rule-in test	49
Commercial NAATs meta-analysis	64	98	it requires DNA isolation and amplification steps, time-consuming with suboptimal sensitivity	50
Nucleic acid commercial amplification tests meta-analysis	56	98		51
In-house qPCR	100 (definite TBM) 98 (probable and possible TBM)	96–97 (definite TBM), 98 (probable and possible TBM)	it requires DNA isolation and amplification steps and is time-consuming	8
In-house qPCR	87.6 (filtrate), 53.1 (sediment)	92		52
Antigen Detection Tests				
MS (current study)	97.44	97.96	excellent diagnostic tool to detect TBM with high accuracy	–
HspX (aptamer-based ALISA)	100	91	excellent diagnostic accuracy, laboratory-based format	40
GlcB, HspX, and PstS1 (antibody-based ELISA)	100 (definite TBM)	96–97 (definite TBM)	excellent diagnostic accuracy, laboratory-based format, time-consuming, uses antibody that might have batch-to-batch variation	8
GlcB, HspX, and MPT51 (antibody-based ELISA)	92–95 (probable and possible TBM)	93–96 (probable and possible TBM)		8
LAM, Ag85 complex, 65 kDa and ESAT-6 (antibody-based ELISA)	87	84	laboratory-based format, time-consuming, uses antibody that might have batch-to-batch variation	53

NAATs, Nucleic Acid Amplification Tests.

spectrum of sample containing DNA aptamer or DNA aptamer + MS. In each case, three scans were recorded in a range of 200–340 nm, and the average of three scans was plotted to examine the target-dependent change in aptamer structure.

Surface Plasmon Resonance (SPR) Analysis of Aptamer Interactions with MS

The binding affinity of ssDNA aptamers to MS was investigated using SPR spectroscopy, where MS was immobilized on a CM5 sensor chip (GE Healthcare). In brief, the carboxylic group on the sensor chip was activated with 1-ethyl-3-(3-dimethylaminopropyl) carbodiimide (EDC) and *N*-hydroxysuccinimide (NHS) (BIAcore, Separations Scientific). Using EDC-NHS chemistry, MS was coupled to the flow cell, and the remaining carboxy site on the chip surface was blocked using 1 M ethanolamine.[42] MS-specific ssDNA aptamer at different concentrations (25, 50, 100, 200, 400, and 800 nM) was injected over the activated flow cells at a flow rate of 10 μL/min for 5 min with a dissociation time of 5 min using BIAcore 3000 instrument (GE Healthcare). The blank surface of the CM5 sensor chip was used for background subtraction. The results were obtained and analyzed using BIAevaluation Software (version 4.1; BIAcore) to determine the equilibrium dissociation constant, K_D, for each aptamer.

Aptamer-Based Inhibition of Catalytic Activity of MS

MS inhibition assay was performed at RT in a 96-well Corning UV-Transparent microplate (Corning, USA).The reaction mixture consisted of 20 mM Tricine buffer (pH 7.5), 5 mM $MgCl_2$, 0.8 mM EDTA, 2 mM glyoxylate, and 2 mM acetyl-CoA, and 0.5 μg purified MS protein was added and incubated at RT for 30 min. The reaction was stopped by the addition of 5,5′-dithiobis-2-nitrobenzoic acid (DTNB) to a final concentration of 2 mM in Tris-HCl (pH 8.0). The amount of CoA-SH released was measured by titrating the free thiol groups with DTNB and measuring the change in absorption at 412 nm.[10] Enzyme activity unit was represented as micromoles per minute (μmol/min). The screening of ssDNA aptamers inhibiting MS enzyme activity was performed by using the same protocol mentioned above with a slight modification. DNA aptamers (2 μM final concentration) and purified MS protein (0.5 μg) were pre-incubated for 30 min in buffer containing 20 mM Tricine buffer (pH 7.5), 5 mM $MgCl_2$, and 0.8 mM EDTA, followed by the addition of 2 mM glyoxylate and 2 mM acetyl-CoA. The enzyme activity in the presence of aptamer was determined as stated above.

Post-SELEX Optimization of Aptamer MS10

Post-SELEX truncation and mutation studies were performed with the best-performing aptamer MS10 to determine the minimal functional unit within it. For this, the MS10 sequence was analyzed using a web-based tool QGRS mapper (http://bioinformatics.ramapo.edu/QGRS/index.php) and G4IPDB (http://bsbe.iiti.ac.in/bsbe/ipdb/index.php) to determine the high-propensity G4-forming region.[43,44] Based on the output, truncated and mutant aptamers were designed and procured from IDT.

Determination of IC_{50} and Inhibitor Constant (K_i) of Aptamer Candidates

The inhibitory activity of aptamers was assessed by determining IC_{50} values for aptamer-mediated inhibition of enzymatic activity of MS. In this assay, 2–10 mM glyoxylate and 2 mM acetyl-CoA were used in the reaction. Purified MS protein (0.5 μg) was incubated with aptamers for 30 min at RT followed by the addition of glyoxylate and acetyl-CoA, and measurement of enzyme activity. Also, MS protein was incubated with substrate for 30 min at RT followed by addition of aptamer and measurement of enzyme activity. The IC_{50} values for individual aptamer inhibition were determined by fitting the data into a sigmoidal dose-response curve in GraphPad Prism software (version 5.01). All of the kinetic measurements were performed at RT. The mode of enzyme inhibition was measured as a function of substrate concentration at a fixed concentration of aptamer, and obtained data were plotted using a double reciprocal Lineweaver-Burk plot. K_i values were determined from linear replots of Lineweaver-Burk slopes versus aptamer or mentioned inhibitor concentrations. Experimental data were analyzed using GraphPad Prism (version 5.01).

Aptamer Binding to Mtb Cell Surface

Log phase 10^7 colony-forming units (CFUs) of Mtb H37Rv was treated with 10 mM vitamin C for 24 h to overexpress the glyoxylate shunt enzymes.[45,46] Cultures were washed with SB and incubated with 0.5 μM 5′-FAM-labeled aptamers (C2 and MS10-Trunc) for 1 h at RT. After removal of unbound aptamers, bacilli were washed, fixed in 2% paraformaldehyde at 4°C for 4 h, and acquired on BD LSRII flow cytometer, to assess aptamer binding under FITC-A channel. Overlay plots were prepared, and data were analyzed using FlowJo 10.4.1 software.

Inhibition of Mtb Invasion into Cells

Mtb H37Rv was cultured in DTA medium (Dubos containing 0.5% BSA, 0.75% dextrose, and 0.085% NaCl plus 0.1% Tween 80) with shaking at 220 rpm at 37°C until OD_{595} ∼0.1–0.2 and treated with 10 mM vitamin C for 24 h. The bacteria were washed with SB and then exposed to 8 μg aptamer (∼2.2 μM) for 1 h in SB. Bacteria were washed to remove excess of aptamer and infected into 5×10^5 THP-1 cells at a MOI of 10:1 for 2 h. The extracellular Mtb were removed by treatment with amikacin (200 μg/mL) for 2 h, after which the Mtb-infected THP-1 cells were lysed with 0.025% SDS. Bacterial CFUs were enumerated by plating THP-1 lysates on Middlebrook 7H11 agar supplemented with 1.5% glycerol (Middlebrook 7H10 agar), and the counts were finalized at 5 weeks.

Diagnostic Application of MS10-Trunc

The diagnostic utility of MS10-Trunc aptamer was evaluated through ALISA on 86 archived cerebrospinal fluid samples.[8,40] The samples belonged to pediatric TBM subjects (n = 38) and disease controls subjects (n = 48). The ALISA procedure was the same as described above except the CSF samples were diluted 1:20 in coating buffer. Data were plotted after background subtraction: (OD_{450} of CSF sample) − (OD_{450} of mean + 3 SD of control wells).

Structure Determination by NMR

To validate the aptamer structure, we acquired ^{1}H-NMR spectra on AVANCE 500 MHz BioSpin International (Switzerland) equipped with a 5-mm broadband inverse probe. All of the NMR spectra were acquired using solvent containing H_2O/D_2O at 9:1 ratio, and spectral data were processed, integrated, and analyzed on Topspin (1.3 version) software. NMR samples were referenced with 3-(trimethylsilyl) propionic-2, 2, 3, 3-d_4 acid sodium salt (TSP).

Molecular Modeling of MS10-Trunc and Docking on MS

To simulate the structure and binding of MS10-Trunc aptamer (5′-GGTGGTGGTGG-3′) to MS, a model of aptamer structure was first generated and used as the input for molecular docking on MS (PDB: 2GQ3). A well-known intra-molecular G4 formed by the SV40 repeat sequence (PDB: 1EVO) was taken as the initial input for modeling the 3D structure of MS10-Trunc aptamer, and further needful mutations were performed on Discovery studio 3.5. Protein 1EVO was chosen as the initial input model because of the presence of a similar kind of G4 topology and close sequence homology between 1EVO and MS10-Trunc aptamer. The resulting structure was subjected to energy minimization and optimized using various iterations of conjugate gradient and steepest descent method. Electrostatics was set as ‘particle mesh Ewald,’ and apply SHAKE constraints were chosen for every minimization run. The molecular docking studies were carried out using Hex 8.0.0 tool in which G4 DNA was treated as a rigid body. Correlation type was set as “Shape+Electro,” and FFT mode was set as 3D for every docking iteration. Sampling methods were set as “Rang angles,” and docking output was ranked according to their free energy.

SUPPLEMENTAL INFORMATION

Supplemental Information can be found online at https://doi.org/10.1016/j.omtn.2019.09.026.

AUTHOR CONTRIBUTIONS

J.S.T., T.K.S., A.K., and T.P.S. designed and supervised the experiment. A.D., C.K., K.S., I.D., S.K.M., and P.S. performed experiments, J.S.T., T.K.S., A.K., A.D., C.K., K.S., I.D., S.K.M., and P.S. collected and analyzed the data. J.S.T., T.K.S., A.D., C.K., K.S., S.K.M., and A.K. wrote the manuscript. All authors reviewed the manuscript.

CONFLICTS OF INTEREST

The authors declare no competing interests.

ACKNOWLEDGMENTS

This work was supported through the National Biodesign Alliance project by Department of Biotechnology, Government of India awarded to J.S.T. (grant BT/PR14638/MED/12/483/2010) and an Innovation Award and THSTI core grant to T.K.S. by the Department of Biotechnology, Government of India. J.S.T. is thankful to the Department of Science and Technology, Government of India for the J. C. Bose National Fellowship during part of the study. T.K.S. is thankful to the Department of Biotechnology, Government of India for the Innovative Young Biotechnologist Award (BT/010/IYBA/

2016/10). A.D. acknowledges the Indian Council of Medical Research, Government of India for a Senior Research Fellowship. K.S. is thankful to Department of Science and Technology, Government of India (grant PDF/2016/000745) for funding. The aptamer sequences reported in this study are proprietary reagents and are deposited in the Indian Patent Office as part of Indian Patent application no. 201611021901. We thank Dr. Priya Kalra for her help during the FACS experiment. The admirable technical assistance of Sanjay Kumar and Jitendra Singh is duly acknowledged. We sincerely thank Sarin Prakash and Sunil Kumar Gupta for their active involvement in data management and sample collection. BEI Resources and NIAID, NIH are acknowledged as below for providing various proteins that were utilized in this study. The following reagents were obtained through BEI Resources and NIAID, NIH: Plasmid pMRLB.8 Containing Gene Rv1837c (Protein GlcB), from *Mycobacterium tuberculosis*, NR-13281; Plasmid pMRLB.38 Containing Gene Rv3803c (Protein Mpt51) from *Mycobacterium tuberculosis*, NR-13291; Plasmid pMRLB.12 Containing Gene Rv1980c (Protein mpt64) from *Mycobacterium tuberculosis*, NR-13273; Plasmid pMRLB.46 Containing Gene Rv3874 (Protein Cfp10) from *Mycobacterium tuberculosis*, NR-13297 and Ag85 Complex; Purified Native Protein from *Mycobacterium tuberculosis*, Strain H37Rv, NR-14855, HspX; Recombinant Protein Reference Standard, NR-49428, GroES (Gene Rv3418c); Purified Native Protein from *Mycobacterium tuberculosis*, Strain H37Rv, NR-14861, *Mycobacterium tuberculosis*, Strain H37Rv; Purified Lipoarabinomannan (LAM), ESAT-6; Recombinant Protein Reference Standard, NR-49424; and *Mycobacterium tuberculosis*, Strain H37Rv, Culture Filtrate Proteins, NR-14825.

REFERENCES

1. World Health Organization (2018). WHO Global TB report, https://www.aidsdatahub.org/global-tuberculosis-report-2018-who-2018.
2. World Health Organization (2017). Global Tuberculosis Report, http://apps.who.int/medicinedocs/en/m/abstract/Js23360en/.
3. Baig, I.A., Moon, J.Y., Lee, S.C., Ryoo, S.W., and Yoon, M.Y. (2015). Development of ssDNA aptamers as potent inhibitors of Mycobacterium tuberculosis acetohydroxyacid synthase. Biochim. Biophys. Acta *1854* (10 pt A), 1338–1350.
4. Shum, K.T., Lui, E.L., Wong, S.C., Yeung, P., Sam, L., Wang, Y., Watt, R.M., and Tanner, J.A. (2011). Aptamer-mediated inhibition of Mycobacterium tuberculosis polyphosphate kinase 2. Biochemistry *50*, 3261–3271.
5. Myler, P.J., and Stacy, R. (2012). A new drug for an old bug. Chem. Biol. *19*, 1499–1500.
6. Marrero, J., Rhee, K.Y., Schnappinger, D., Pethe, K., and Ehrt, S. (2010). Gluconeogenic carbon flow of tricarboxylic acid cycle intermediates is critical for Mycobacterium tuberculosis to establish and maintain infection. Proc. Natl. Acad. Sci. USA *107*, 9819–9824.
7. Kinhikar, A.G., Vargas, D., Li, H., Mahaffey, S.B., Hinds, L., Belisle, J.T., and Laal, S. (2006). Mycobacterium tuberculosis malate synthase is a laminin-binding adhesin. Mol. Microbiol. *60*, 999–1013.
8. Haldar, S., Sankhyan, N., Sharma, N., Bansal, A., Jain, V., Gupta, V.K., Juneja, M., Mishra, D., Kapil, A., Singh, U.B., et al. (2012). Detection of Mycobacterium tuberculosis GlcB or HspX Antigens or devR DNA impacts the rapid diagnosis of tuberculous meningitis in children. PLoS ONE *7*, e44630.
9. Sharma, V., Sharma, S., Hoener zu Bentrup, K., McKinney, J.D., Russell, D.G., Jacobs, W.R., Jr., and Sacchettini, J.C. (2000). Structure of isocitrate lyase, a persistence factor of Mycobacterium tuberculosis. Nat. Struct. Biol. *7*, 663–668.
10. Smith, C.V., Huang, C.C., Miczak, A., Russell, D.G., Sacchettini, J.C., and Höner zu Bentrup, K. (2003). Biochemical and structural studies of malate synthase from Mycobacterium tuberculosis. J. Biol. Chem. *278*, 1735–1743.
11. Puckett, S., Trujillo, C., Wang, Z., Eoh, H., Ioerger, T.R., Krieger, I., Sacchettini, J., Schnappinger, D., Rhee, K.Y., and Ehrt, S. (2017). Glyoxylate detoxification is an essential function of malate synthase required for carbon assimilation in *Mycobacterium tuberculosis*. Proc. Natl. Acad. Sci. USA *114*, E2225–E2232.
12. Sobotka, O., Endlicher, R., Drahota, Z., Kučera, O., Rychtrmoc, D., Raad, M., Hakeem, K., and Červinková, Z. (2016). Impaired mitochondrial functions contribute to 3-bromopyruvate toxicity in primary rat and mouse hepatocytes. J. Bioenerg. Biomembr. *48*, 363–373.
13. Dunn, M.F., Ramírez-Trujillo, J.A., and Hernández-Lucas, I. (2009). Major roles of isocitrate lyase and malate synthase in bacterial and fungal pathogenesis. Microbiology *155*, 3166–3175.
14. Krieger, I.V., Freundlich, J.S., Gawandi, V.B., Roberts, J.P., Gawandi, V.B., Sun, Q., Owen, J.L., Fraile, M.T., Huss, S.I., Lavandera, J.L., et al. (2012). Structure-guided discovery of phenyl-diketo acids as potent inhibitors of M. tuberculosis malate synthase. Chem. Biol. *19*, 1556–1567.
15. Huang, H.L., Krieger, I.V., Parai, M.K., Gawandi, V.B., and Sacchettini, J.C. (2016). Mycobacterium tuberculosis Malate Synthase Structures with Fragments Reveal a Portal for Substrate/Product Exchange. J. Biol. Chem. *291*, 27421–27432.
16. Kedzierski, S., Khoshnejad, M., and Caltagirone, G.T. (2013). Synthetic antibodies: the emerging field of aptamers. Bioprocess. J. *12*, 46–49.
17. Kaur, H., Bruno, J.G., Kumar, A., and Sharma, T.K. (2018). Aptamers in the Therapeutics and Diagnostics Pipelines. Theranostics *8*, 4016–4032.
18. Hamula, C.L., Le, X.C., and Li, X.F. (2011). DNA aptamers binding to multiple prevalent M-types of Streptococcus pyogenes. Anal. Chem. *83*, 3640–3647.
19. Nagatoishi, S., Isono, N., Tsumoto, K., and Sugimoto, N. (2011). Loop residues of thrombin-binding DNA aptamer impact G-quadruplex stability and thrombin binding. Biochimie *93*, 1231–1238.
20. Phan, A.T., Kuryavyi, V., Ma, J.B., Faure, A., Andréola, M.L., and Patel, D.J. (2005). An interlocked dimeric parallel-stranded DNA quadruplex: a potent inhibitor of HIV-1 integrase. Proc. Natl. Acad. Sci. USA *102*, 634–639.
21. Platella, C., Riccardi, C., Montesarchio, D., Roviello, G.N., and Musumeci, D. (2017). G-quadruplex-based aptamers against protein targets in therapy and diagnostics. Biochim. Biophys. Acta, Gen. Subj. *1861* (5 pt B), 1429–1447.
22. Sharma, T.K., Bruno, J.G., and Dhiman, A. (2017). ABCs of DNA aptamer and related assay development. Biotechnol. Adv. *35*, 275–301.
23. Kypr, J., Kejnovská, I., Renciuk, D., and Vorlícková, M. (2009). Circular dichroism and conformational polymorphism of DNA. Nucleic Acids Res. *37*, 1713–1725.
24. Choi, E.W., Nayak, L.V., and Bates, P.J. (2010). Cancer-selective antiproliferative activity is a general property of some G-rich oligodeoxynucleotides. Nucleic Acids Res. *38*, 1623–1635.
25. Tucker, W.O., Shum, K.T., and Tanner, J.A. (2012). G-quadruplex DNA aptamers and their ligands: structure, function and application. Curr. Pharm. Des. *18*, 2014–2026.
26. He, X., Guo, L., He, J., Xu, H., and Xie, J. (2017). Stepping Library-Based Post-SELEX Strategy Approaching to the Minimized Aptamer in SPR. Anal. Chem. *89*, 6559–6566.
27. Tawani, A., Amanullah, A., Mishra, A., and Kumar, A. (2016). Evidences for Piperine inhibiting cancer by targeting human G-quadruplex DNA sequences. Sci. Rep. *6*, 39239.
28. Kondrashov, F.A., Koonin, E.V., Morgunov, I.G., Finogenova, T.V., and Kondrashova, M.N. (2006). Evolution of glyoxylate cycle enzymes in Metazoa: evidence of multiple horizontal transfer events and pseudogene formation. Biol. Direct *1*, 31.
29. Keefe, A.D., Pai, S., and Ellington, A. (2010). Aptamers as therapeutics. Nat. Rev. Drug Discov. *9*, 537–550.
30. Nimjee, S.M., White, R.R., Becker, R.C., and Sullenger, B.A. (2017). Aptamers as Therapeutics. Annu. Rev. Pharmacol. Toxicol. *57*, 61–79.

31. Zhou, J., and Rossi, J. (2017). Aptamers as targeted therapeutics: current potential and challenges. Nat. Rev. Drug Discov. *16*, 440.

32. Macdonald, J., Houghton, P., Xiang, D., Duan, W., and Shigdar, S. (2016). Truncation and Mutation of a Transferrin Receptor Aptamer Enhances Binding Affinity. Nucleic Acid Ther. *26*, 348–354.

33. Bates, P.J., Laber, D.A., Miller, D.M., Thomas, S.D., and Trent, J.O. (2009). Discovery and development of the G-rich oligonucleotide AS1411 as a novel treatment for cancer. Exp. Mol. Pathol. *86*, 151–164.

34. Nadal, P., Svobodova, M., Mairal, T., and O'Sullivan, C.K. (2013). Probing high-affinity 11-mer DNA aptamer against Lup an 1 (β-conglutin). Anal. Bioanal. Chem. *405*, 9343–9349.

35. Hotoda, H., Koizumi, M., Koga, R., Kaneko, M., Momota, K., Ohmine, T., Furukawa, H., Agatsuma, T., Nishigaki, T., Sone, J., et al. (1998). Biologically active oligodeoxyribonucleotides. 5. 5′-End-substituted d(TGGGAG) possesses anti-human immunodeficiency virus type 1 activity by forming a G-quadruplex structure. J. Med. Chem. *41*, 3655–3663.

36. Squeglia, F., Ruggiero, A., De Simone, A., and Berisio, R. (2018). A structural overview of mycobacterial adhesins: Key biomarkers for diagnostics and therapeutics. Protein Sci. *27*, 369–380.

37. Chen, F., Zhang, X., Zhou, J., Liu, S., and Liu, J. (2012). Aptamer inhibits Mycobacterium tuberculosis (H37Rv) invasion of macrophage. Mol. Biol. Rep. *39*, 2157–2162.

38. Liao, J., Liu, B., Liu, J., Zhang, J., Chen, K., and Liu, H. (2015). Cell-specific aptamers and their conjugation with nanomaterials for targeted drug delivery. Expert Opin. Drug Deliv. *12*, 493–506.

39. AlShamaileh, H., and Veedu, R.N. (2017). Next-Generation Nucleic Acid Aptamers with Two-Base-Modified Nucleotides Have Improved Binding Affinity and Potency. ChemBioChem *18*, 1565–1567.

40. Dhiman, A., Haldar, S., Mishra, S.K., Sharma, N., Bansal, A., Ahmad, Y., Kumar, A., Sharma, T.K., and Tyagi, J.S. (2018). Generation and application of DNA aptamers against HspX for accurate diagnosis of tuberculous meningitis. Tuberculosis (Edinb.) *112*, 27–36.

41. Kalra, P., Mishra, S.K., Kaur, S., Kumar, A., Prasad, H.K., Sharma, T.K., and Tyagi, J.S. (2018). G-Quadruplex-Forming DNA Aptamers Inhibit the DNA-Binding Function of HupB and Mycobacterium tuberculosis Entry into Host Cells. Mol. Ther. Nucleic Acids *13*, 99–109.

42. Rotherham, L.S., Maserumule, C., Dheda, K., Theron, J., and Khati, M. (2012). Selection and application of ssDNA aptamers to detect active TB from sputum samples. PLoS ONE *7*, e46862.

43. Kikin, O., D'Antonio, L., and Bagga, P.S. (2006). QGRS Mapper: a web-based server for predicting G-quadruplexes in nucleotide sequences. Nucleic Acids Res. *34*, W676–W682.

44. Mishra, S.K., Tawani, A., Mishra, A., and Kumar, A. (2016). G4IPDB: A database for G-quadruplex structure forming nucleic acid interacting proteins. Sci. Rep. *6*, 38144.

45. Sikri, K., Duggal, P., Kumar, C., Batra, S.D., Vashist, A., Bhaskar, A., Tripathi, K., Sethi, T., Singh, A., and Tyagi, J.S. (2018). Multifaceted remodeling by vitamin C boosts sensitivity of Mycobacterium tuberculosis subpopulations to combination treatment by anti-tubercular drugs. Redox Biol. *15*, 452–466.

46. Taneja, N.K., Dhingra, S., Mittal, A., Naresh, M., and Tyagi, J.S. (2010). Mycobacterium tuberculosis transcriptional adaptation, growth arrest and dormancy phenotype development is triggered by vitamin C. PLoS ONE *5*, e10860.

47. Thakur, R., Goyal, R., and Sarma, S. (2010). Laboratory diagnosis of tuberculous meningitis—is there a scope for further improvement? J. Lab. Physicians *2*, 21–24.

48. Thwaites, G., Chau, T.T., Mai, N.T., Drobniewski, F., McAdam, K., and Farrar, J. (2000). Tuberculous meningitis. J. Neurol. Neurosurg. Psychiatry *68*, 289–299.

49. Rufai, S.B., Singh, A., Singh, J., Kumar, P., Sankar, M.M., and Singh, S.; TB Research Team (2017). Diagnostic usefulness of Xpert MTB/RIF assay for detection of tuberculous meningitis using cerebrospinal fluid. J. Infect. *75*, 125–131.

50. Solomons, R.S., van Elsland, S.L., Visser, D.H., Hoek, K.G., Marais, B.J., Schoeman, J.F., and van Furth, A.M. (2014). Commercial nucleic acid amplification tests in tuberculous meningitis—a meta-analysis. Diagn. Microbiol. Infect. Dis. *78*, 398–403.

51. Pai, M., Flores, L.L., Pai, N., Hubbard, A., Riley, L.W., and Colford, J.M., Jr. (2003). Diagnostic accuracy of nucleic acid amplification tests for tuberculous meningitis: a systematic review and meta-analysis. Lancet Infect. Dis. *3*, 633–643.

52. Haldar, S., Sharma, N., Gupta, V.K., and Tyagi, J.S. (2009). Efficient diagnosis of tuberculous meningitis by detection of Mycobacterium tuberculosis DNA in cerebrospinal fluid filtrates using PCR. J. Med. Microbiol. *58*, 616–624.

53. Flores, L.L., Steingart, K.R., Dendukuri, N., Schiller, I., Minion, J., Pai, M., Ramsay, A., Henry, M., and Laal, S. (2011). Systematic review and meta-analysis of antigen detection tests for the diagnosis of tuberculosis. Clin. Vaccine Immunol. *18*, 1616–1627.

OMTN, Volume *18*

Supplemental Information

Theranostic Application of a Novel G-Quadruplex-Forming DNA Aptamer Targeting Malate Synthase of Mycobacterium tuberculosis

Abhijeet Dhiman, Chanchal Kumar, Subodh Kumar Mishra, Kriti Sikri, Ishara Datta, Pradeep Sharma, Tej P. Singh, Sagarika Haldar, Neera Sharma, Anjali Bansal, Yusra Ahmad, Amit Kumar, Tarun Kumar Sharma, and Jaya Sivaswami Tyagi

SUPPLEMENTARY FIGURELEGENDS

Figure S1

Sequence alignment based on primary sequence homology reveals two categories i.e. G-rich and non-G rich group.

Figure S2

Base fraction analysis of selected aptamer candidates showing abundance of various base fractions (A, T, G or C), 50% of aptamer pool represents G-richness **(A)**and abundance of G+C and A+T **(B).**

Figure S3

Secondary structure of selected aptamer candidates predicted by web-based tool RNA-fold (http://rna.tbi.univie.ac.at/cgi-bin/RNAWebSuite/RNAfold.cgi) using DNA parameters.

Figure S4

The SPR-sensograms for the bindings of ssDNA aptamers namely, MS4, MS5, MS6, MS10, MS20, MS10-Trunc and R0 (Naïve library) with malate synthase (MS). The protein MS was immobilized on the CM5sensor chip and the increasing concentrations (25, 50, 100, 200, 400 and 800 nM) of ssDNA aptamers were used in mobile phase in separate experiments.

Figure S5

Secondary structure of MS10 and its truncated form MS10-Trunc predicted by web-based tool RNA-fold (http://rna.tbi.univie.ac.at/cgi-bin/RNAWebSuite/RNAfold.cgi).

Figure S6

MS10-Trunc aptamer-based detection of Mtb MS in CSF specimens by ALISA. **(A)** Scatter plot of ΔOD450 value was generated from ‘Definite TB’ (True positive) vs. NTIM group (True negative). *** represent statistical significance at p value of <0.0001. **(B)** ROC curve generated from ‘Definite TB’ vs. ‘NTIM group’. The blue horizontal line across the scatter plot indicates the cut-off (0.3444) derived by ROC curve analysis of ‘Definite TB’ and NTIM disease specimens.

Classification of cerebrospinal fluid (CSF) samples

A subset of 86 pediatric CSF samples that was derived from an archived set of samples described previously by Haldar et al[1] was used in the present study. The samples were categorized according to a universal case definition for tuberculous meningitis (TBM)[2]. Culture/smear/commercial nucleic acid amplification test (NAAT) positive/ acid-fast bacillus (AFB) seen on autopsy were categorized as "definite" TBM. "Probable" and "possible" TBM groups include subjects negative by the above criteria but satisfying the defined clinical criteria, CSF biochemistry and cytology, cerebral imaging criteria and evidence of extraneural TB having a score of ≥10–12 ("probable" TBM) and a score of ≥6–11 ("possible" TBM). In this CSF sample subset (N=86), samples were classified as "definite" TBM on the basis of *M. tuberculosis* culture positivity only (N=16) and as "probable" TBM (N=6, score range: 10–18), "possible" TBM (N=16, score range: 6–9) and "not-tuberculous meningitis" (not-TBM) with an alternative diagnosis established (N=48). The "not-TBM" category was further sub-classified into nontuberculous infectious meningitis (NTIM, N=16), infectious neurological disorders (IND, N=17) and noninfectious neurological disorders (NIND, N=15). The NTIM comprised of cases of pyogenic bacterial meningitis that included 2 culture-confirmed cases of *E. coli* (N=1), and *Acinetobacter* sp. (N=1). Another 14 cases were diagnosed on the basis of response to appropriate antibiotics, clinical presentation and symptoms. The IND category included 6 cases of meningoencephalitis, 4 cases of enteric encephalopathy, 3 cases of sepsis, 2 cases of cerebral malaria and 1 case each of pneumonia and post diphtheritic polyneuritis. The NIND category included 8 cases of neurodegenerative disorders, 2 cases each of hypocalcemic seizures and transverse myelitis, 3 case of Guillain–Barré syndrome.

References

1 Haldar, S. *et al.* Detection of Mycobacterium tuberculosis GlcB or HspX Antigens or devR DNA impacts the rapid diagnosis of tuberculous meningitis in children. *PloS one* **7**, e44630, doi:10.1371/journal.pone.0044630 (2012).

2 Marais, S. *et al.* Tuberculous meningitis: a uniform case definition for use in clinical research. *The Lancet. Infectious diseases* **10**, 803-812, doi:10.1016/S1473-3099(10)70138-9 (2010).

3 Phan, A. T. *et al.* An interlocked dimeric parallel-stranded DNA quadruplex: a potent inhibitor of HIV-1 integrase. *Proceedings of the National Academy of Sciences of the United States of America* **102**, 634-639, doi:10.1073/pnas.0406278102 (2005).

4 Nagatoishi, S., Isono, N., Tsumoto, K. & Sugimoto, N. Loop residues of thrombin-binding DNA aptamer impact G-quadruplex stability and thrombin binding. *Biochimie* **93**, 1231-1238, doi:10.1016/j.biochi.2011.03.013 (2011).

5 Platella, C., Riccardi, C., Montesarchio, D., Roviello, G. N. & Musumeci, D. G-quadruplex-based aptamers against protein targets in therapy and diagnostics. *Biochimica et biophysica acta. General subjects* **1861**, 1429-1447, doi:10.1016/j.bbagen.2016.11.027 (2017).

Supplementary Figure S1

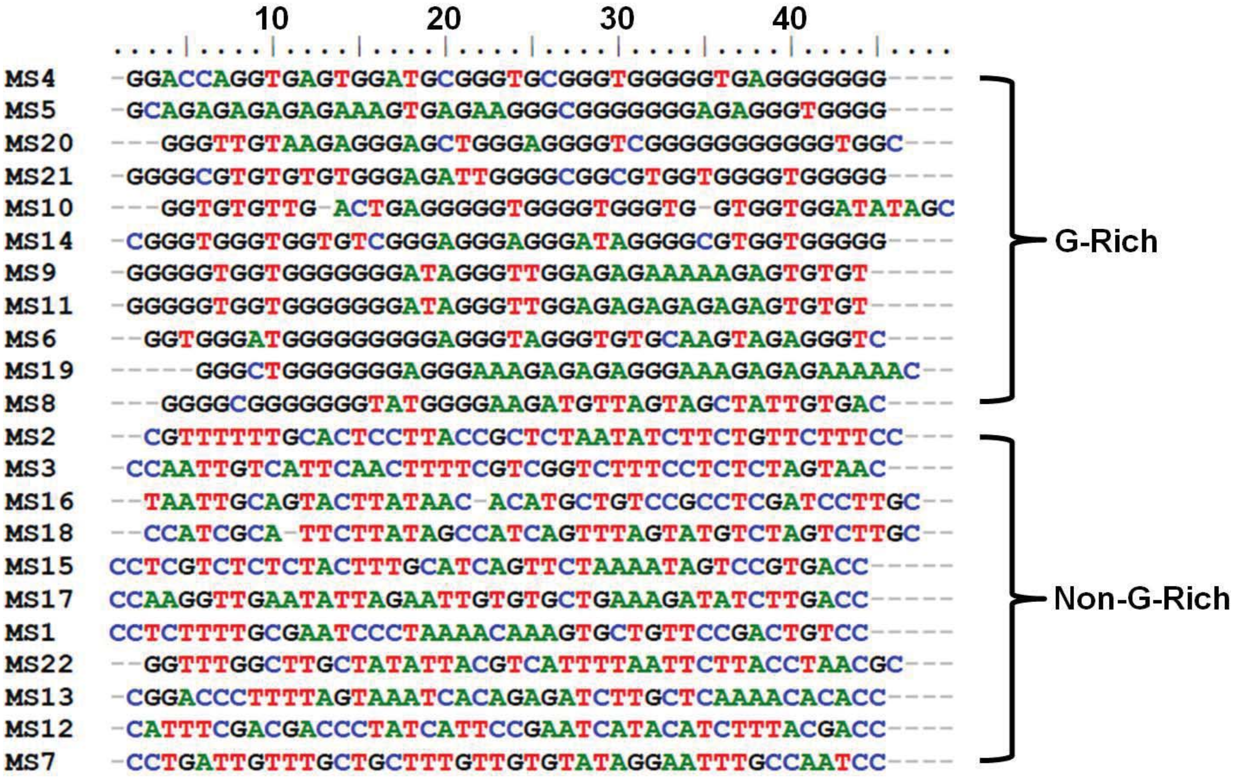

Supplementary Figure S2

A

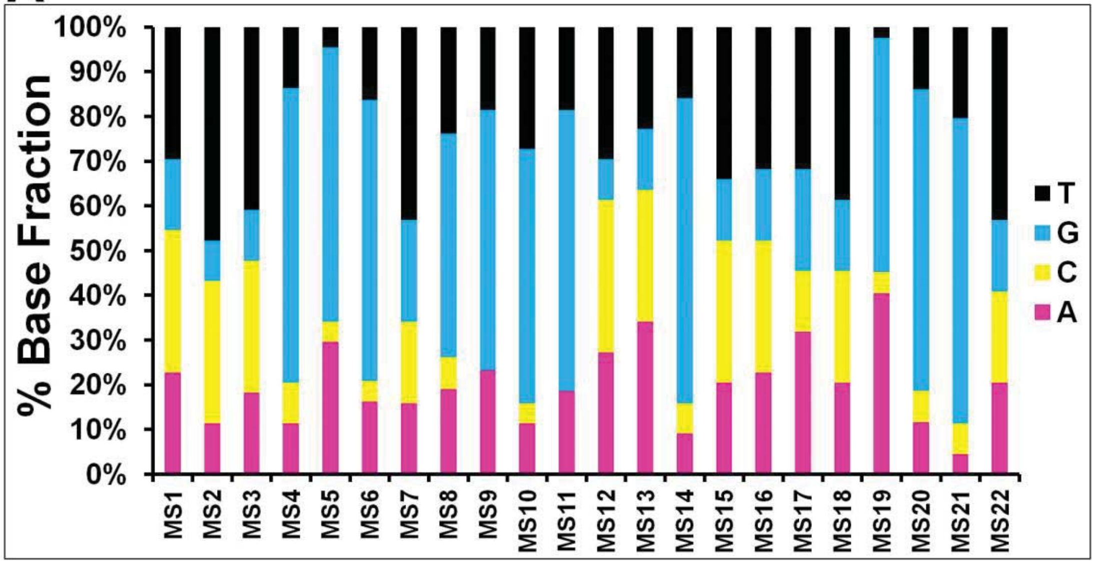

B

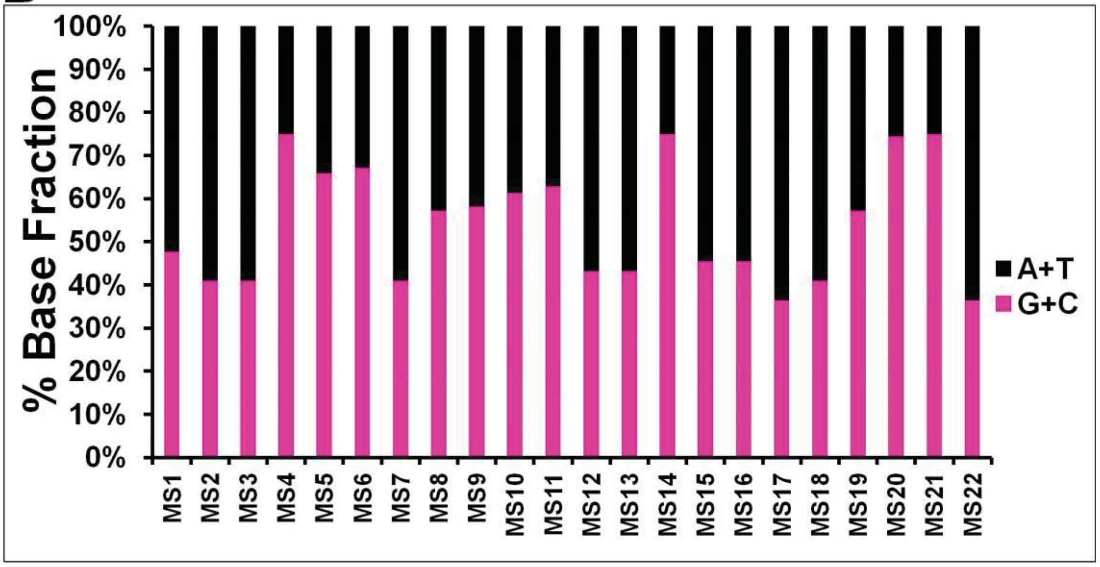

Supplementary Figure S3

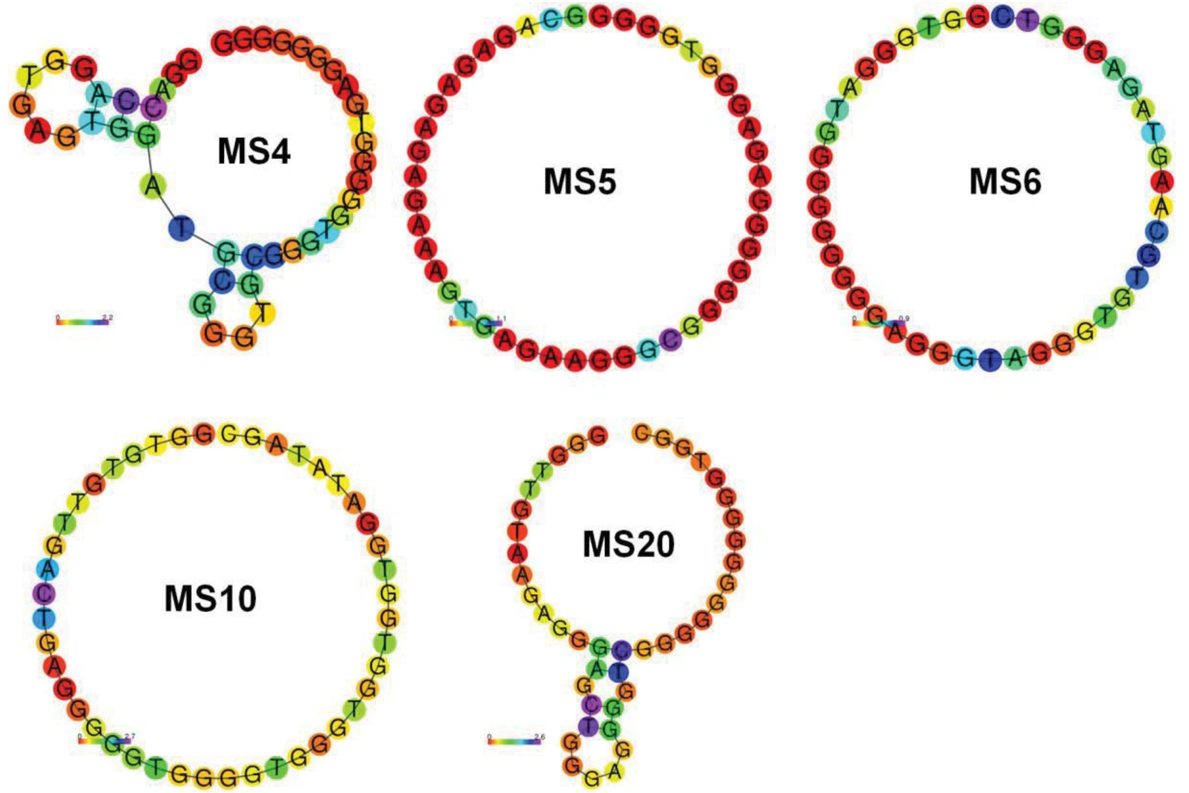

Supplementary Figure S4

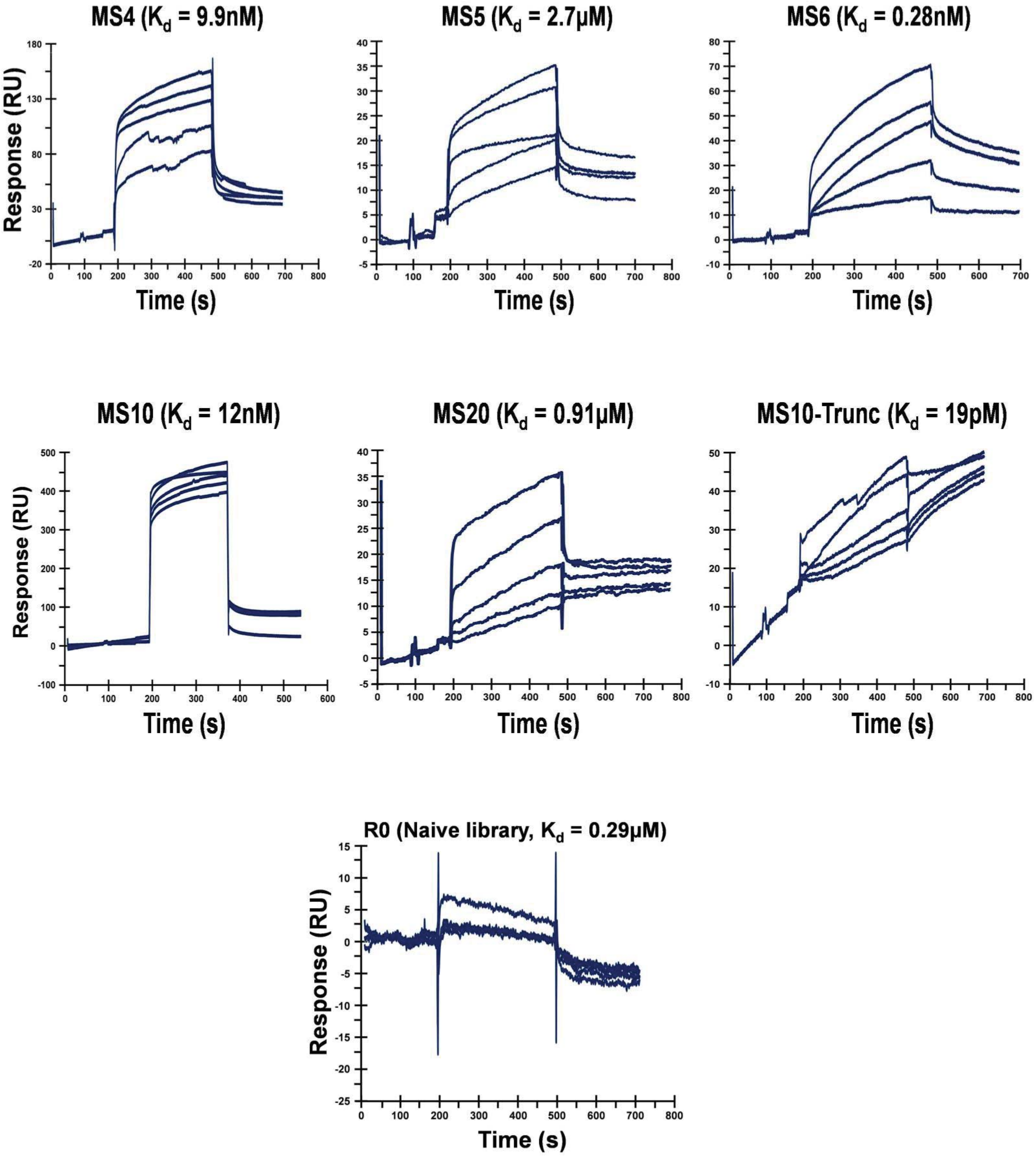

Supplementary Figure S5

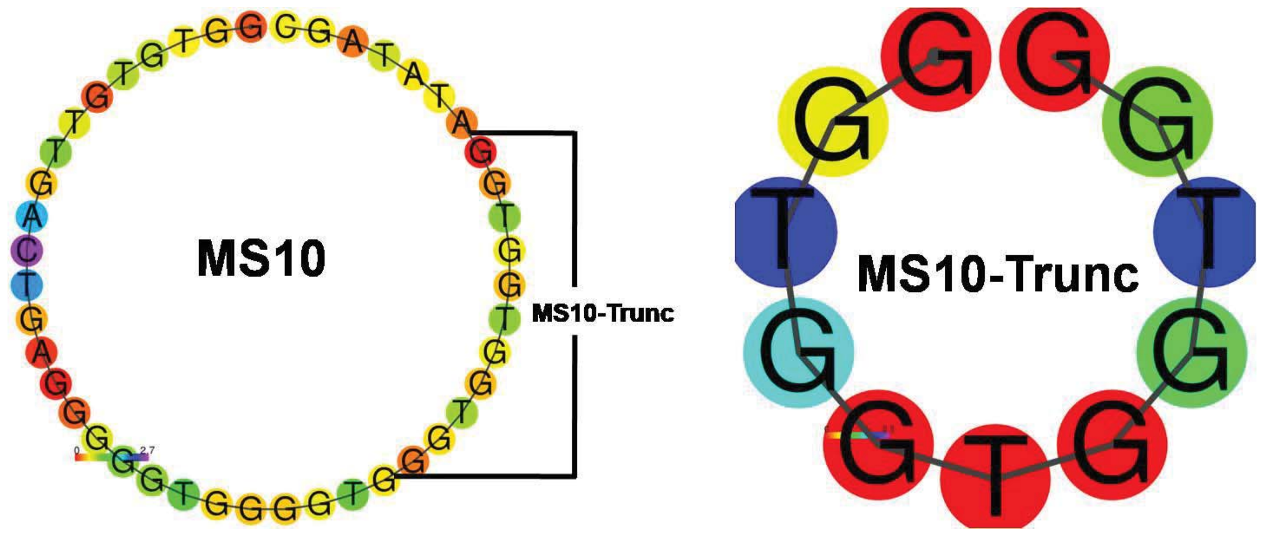

Supplementary Figure S6

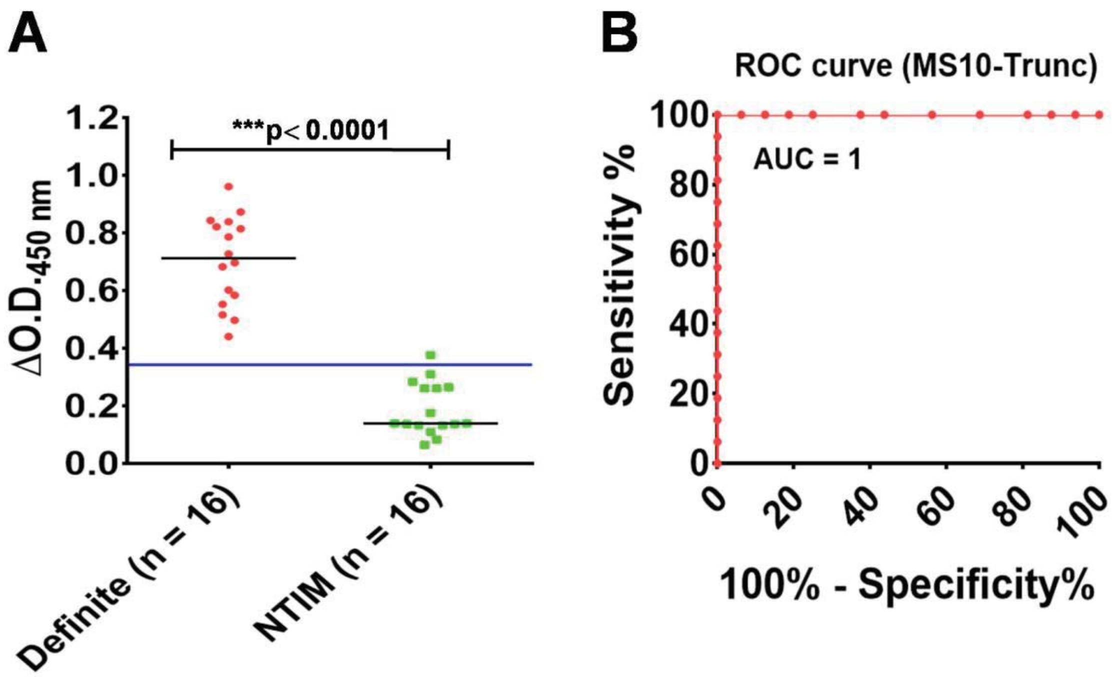

Supplementary Table S1: Sequence of aptamers subjected to QGRS mapper analysis and comparison of their G-scores with those of reported G-quadruplex (G4) forming aptamers.

S.No.	Name	Sequences*	G-Score	Length of G4 forming region (nt)	References
1.	MS4	GGACCAGGTGAGTGGATGC**GGGTGCGGGTGGGGGTGAGGG**GGGG	42	21	This study
2.	MS5	GCAGAGAGAGAGAAAGTGAGAA**GGGCGGGGGGGAGAGGGTGGGG**	40	22	
3.	MS6	GGTGGGAT**GGGGGGGGGAGGGTAGGG**TGTGCAAGTAGAGGGTC	42	18	
4.	MS10	GGTGTGTTGACTGAGGGGGTGGGGTG**GGTGGTGGTGG**ATATAGC	21	11	
5.	MS20	GGGTTGTAAGAGGGAGCT**GGGAGGGGTCGGGGGGGG**GGGTGGC	42	18	
6.	Anti HIV-1 integrase	GGGTGGGAGGAGGG	21	14	3
7.	Thrombin	GGTTGGTGTGGTTGG	20	15	4
8.	Anti-VEGF	GGGGTGGACGGGCCGGG	21	17	5

*** G4 forming regions in the various aptamers are underlined and in red.**

Supplementary Table S2: Primary nucleotide sequence of the control aptamers used in the study.

S.No.	Name	Control Aptamers
1.	C1	5’CCCCGTTGCGTCCCGTCTAGACCTAATATGATCCACGAATTATC3’
2.	C2	5’TTTTTTTTTTTTTTTTTTTTTTTTTTTTTTTTTTTTTTTTTTTT3’

Supplementary Table S3: Nucleotide sequences of MS10 aptamer, its truncated and mutant derivatives. Bold nucleotides represent the truncated portion of aptamer MS10. Red highlighted base represent the site of mutation (deletion).

S.No.	Aptamer Name	Sequence
1.	MS10	GGTGTGTTGACTGAGGGGGTGGGGTG**GGTGGTGGTGG**ATATAGC
2.	MS10-Trunc	**GGTGGTGGTGG**
3.	MS10-Trunc ΔT3	**GGTGGTGGTGG**
4.	MS10-TruncΔT6	**GGTGGTGGTGG**
5.	MS10- Trunc ΔT9	**GGTGGTGGTGG**
